# Evaluating the strength properties of high-performance concrete in the form of ensemble and hybrid models using deep learning techniques

**DOI:** 10.1038/s41598-025-10860-y

**Published:** 2025-07-15

**Authors:** Zhe Wang, Tao Sun, Yan Sun, Na Liu

**Affiliations:** 1 National Center for Nanoscience and Technology, Beijing, 100190 China; 2 Beijing GrandTrend International Economic and Technical Consulting Co., Ltd. , Beijing, 100012 China; 3 Tianjin University Research Institute of Architectural Design and Urban Planning Co., Ltd., Tianjin, 300072 China; 4 Guangzhou Changdi Spatial Information Technology Co., Ltd., Guangzhou, 510663 Guangdong China

**Keywords:** Slump flow, Compressive CS, T-S fuzzy inference system, Gradient boosting machines, Decision tree, Grey wolf optimizer, Engineering, Materials science

## Abstract

In the behavior of concrete, factors such as particle types, water content, aggregates, additives, and binders significantly influence its Compressive Strength (CS) properties. This study develops hybrid and ensemble models to predict compressive CS and slump flow of high-performance concrete (HPC) using a dataset of 191 mixtures. Admixtures like fly ash and silica fume enhance HPC through hydraulic or pozzolanic activity. Understanding the relationships between HPC components is crucial for computational analysis of CS properties. Deep learning techniques, including hybrid and ensemble methods, were developed to predict these properties with high accuracy. This paper focuses on forecasting models using T-SFIS, GBMBoost, and Decision Tree, combined with metaheuristic algorithms (GWO, QPSO) in hybrid and ensemble frameworks. Sensitivity analysis via SHAP and tenfold cross-validation evaluated model performance. Results showed that the GWO-based GBQP model achieved superior performance (${\text{R}}^{2}$=0.998, RMSE = 1.216 MPa for compressive CS). The ensemble DGT model ranked second, while T-SFIS performed lowest. For slump flow, TSQP excelled (${\text{R}}^{2}$=0.984, RMSE = 3.233 mm), closely followed by GBQP. These advanced techniques significantly enhance the efficiency and accuracy of predicting HPC CS properties.

## Introduction

HPC has seen enhancements in recent years. HPC has been employed in the construction sector for specific constructions, including tunnels, nuclear facilities, ports, prefabricated elements, and bridges, owing to its exceptional CS^1,2^. Traditional concrete comprises three fundamental components: fine aggregates, coarse aggregates, and Portland cement, combined with water. In contrast, the manufacturing of HPC necessitates supplementary resources, including blast furnace slag, fly ash, and chemical admixtures like superplasticizers^3^. A primary objective is to accurately assess the intensity based on the mixture ratio^4^. Given that CS is a commonly employed criterion in concrete production, numerous independent research suggest that concrete CS is assessed based on water-to-cement ratios and other components^5^. The application of metaheuristic optimization and machine learning algorithms to forecast concrete’s mechanical characteristics, particularly its compressive CS, has drawn a lot of interest recently. Concrete CS has been successfully predicted by a study that combines new algorithms like krill swarm and leopard jaw with ensemble models like random forest and XGBoost^6^. Additionally, because of its great CS and environmentally friendly approach, ultra-high- CS concrete (UHPC) made from waste materials is seen as a viable option for sustainable building. Its modeling using algorithms like POA and WOA has been studied^7^. Several optimization techniques, including CSA, WS, HH, and FO, have been used to the field of resistant concrete in order to increase the XGB model’s predictive accuracy for CS^8^. The full substitution of fly ash for cement in geopolymer concrete has also been done in accordance with the development of green concretes in order to examine the impact of input parameters on CS^9^. Furthermore, the effective involvement of characteristics like density and recycled materials has been demonstrated by the development of a hybrid model for predicting shear strength (STS) utilizing XGB and algorithms like RS, GS, BO, PSO, and GWO^10^. The influence of metakaolin, silica fume, granulated blast furnace slag, and fly ash on the setting time of high-strength concrete was examined by the penetration resistance method^11–13^. HPC encompasses a diverse array of intricate materials, and simulating their behavior is a challenging task^14^. Forecasting the CS of concrete is a critical subject in concrete construction. Researchers in^15^ have used a 3D mesh-free technique to model the failure of some reinforced concrete (RC) structures. In a study, particle approaches were utilized to address various fracture issues, including reinforced concrete structures, and the computational outcomes shown strong concordance with experimental data^16^. Additionally, another study devised a two-dimensional methodology to evaluate the strength of reinforced concrete structures, taking into account the interaction between the concrete and its CS^17^. in^18,19^ conducted a laboratory investigation on the shrinkage of high-performance concrete including fly ash and ground granulated blast-furnace slag^20^. Moreover, many nonlinear and linear methodologies have been evaluated to ascertain the correlations between the principal factors and aggregate doses that could affect the slump and CS of HPC^21,22^. However, these methods complicate the attainment of precise predictions due to the influence of numerous factors on the Strength qualities of HPC. Properties of HPC, like slump flow and CS, show extremely nonlinear interactions with silica fume, superplasticizer, and water-to-binder ratio, making it difficult for conventional approaches to make accurate forecasts. Through the integration of T-SFIS, GBM, and DT with metaheuristic optimizations (GWO, QPSO), the suggested hybrid and ensemble models (GBQP, TSQP, DGT) successfully capture these complexity by optimizing hyperparameters to model intricate patterns. For instance, GBQP outperformed baseline models like XGBoost and CatBoost, with an R^2^ of 0.998 and an RMSE of 1.226 MPa for CS^23^.

The context for the current investigation comes from a comparison of several studies that have predicted concrete qualities using metaheuristic algorithms and sophisticated machine learning techniques. To forecast the CS of stable concrete, for instance, researchers in^6^ coupled Random Forest and XGBoost models with novel metaheuristic algorithms like krill swarm and leopard jaw. They obtained R^2^ = 0.98, however they noted computational complexity as a drawback. The significance of fine-tuning hyperparameters is further demonstrated by reference^8^, which optimized XGBoost using the CSA, WS, HH, and FO algorithms and obtained R^2^ up to 0.987 for high-strength concrete. The superiority of hybrid approaches was confirmed by Reference^7^, which created a hybrid model using POA and WOA for UHPC concrete and obtained R^2^ between 0.95 and 0.97. Although they acknowledged processing limits for huge data, researchers in^14^ employed SVR with optimization methods and attained R^2^ = 0.94. Additionally, another study^18^ used hybrid approaches to HPC and obtained R2 values ranging from 0.93 to 0.96, demonstrating the benefit of mixing models.

Thus, the standard techniques utilized for traditional concrete frequently prove inadequate for predicting the slump and CS of HPC^24^. In response to advancements in Artificial Intelligence (AI) techniques, numerous researchers are prioritizing soft-based methodologies over expensive experimental experiments. HPC and ultra-high-performance concrete (UHPC) are critical for advanced structural applications due to their superior strength properties^25^. Prasad et al. created an artificial neural network (ANN) model to accurately forecast some Strength characteristics of HPC, demonstrating effective modeling performance^26,27^. AI-based methodologies are significantly dependent on the baseline parameters, imposing a substantial constraint on modeling efficacy. Consequently, the successor must modify the operational procedures of AI models^28^. When compared to single models, the combination and ensemble models increase the prediction accuracy of HPC strength attributes by combining T-SFIS, GBM, and decision trees with metaheuristic algorithms (GWO, QPSO). For instance, Table 3 shows that the combined GBQP model lowers the RMSE by 65% from 3.45 to 1.226 MPa and obtains an R2 of 0.998 for CS, which is an 8% improvement over the regular GBM. By effectively optimizing the aforementioned parameters, these models enhance the simulation process, particularly the computational prediction of CS and slump flow.

This work employed an advanced approach to precisely forecast the HPC Strength characteristics, including CS and slump. The study employs T-S Fuzzy Inference System (T-SFIS)^29^, Gradient Boosting Machines (GBM)^30^, and Decision Tree (DT)^31^ as its machine learning methodologies. T-SFIS, created by in^32^, integrates artificial neural networks with fuzzy logic and employs a data training set akin to that of an artificial neural network to facilitate learning through examples^33^. Moreover, DT, a widely used machine-learning technique, possesses a strong ability to minimize errors in ensemble models using attribute-based (random sub-spaces, RS) and two-instance-based (Bagging and Stochastic Gradient Boosting, GB) features, establishing it as a formidable modeling framework^34,35^. The utilization of GBMBoost was grounded in theoretical principles, whereby the outcome of the boosting algorithm, equipped with a filter framework, can ascertain the limits of the generalization mistake associated with the boosting method. Adaptive control under sensor faults^36^ highlights challenges in ensuring model robustness, similar to limitations in HPC prediction.

**Fig. 1:**
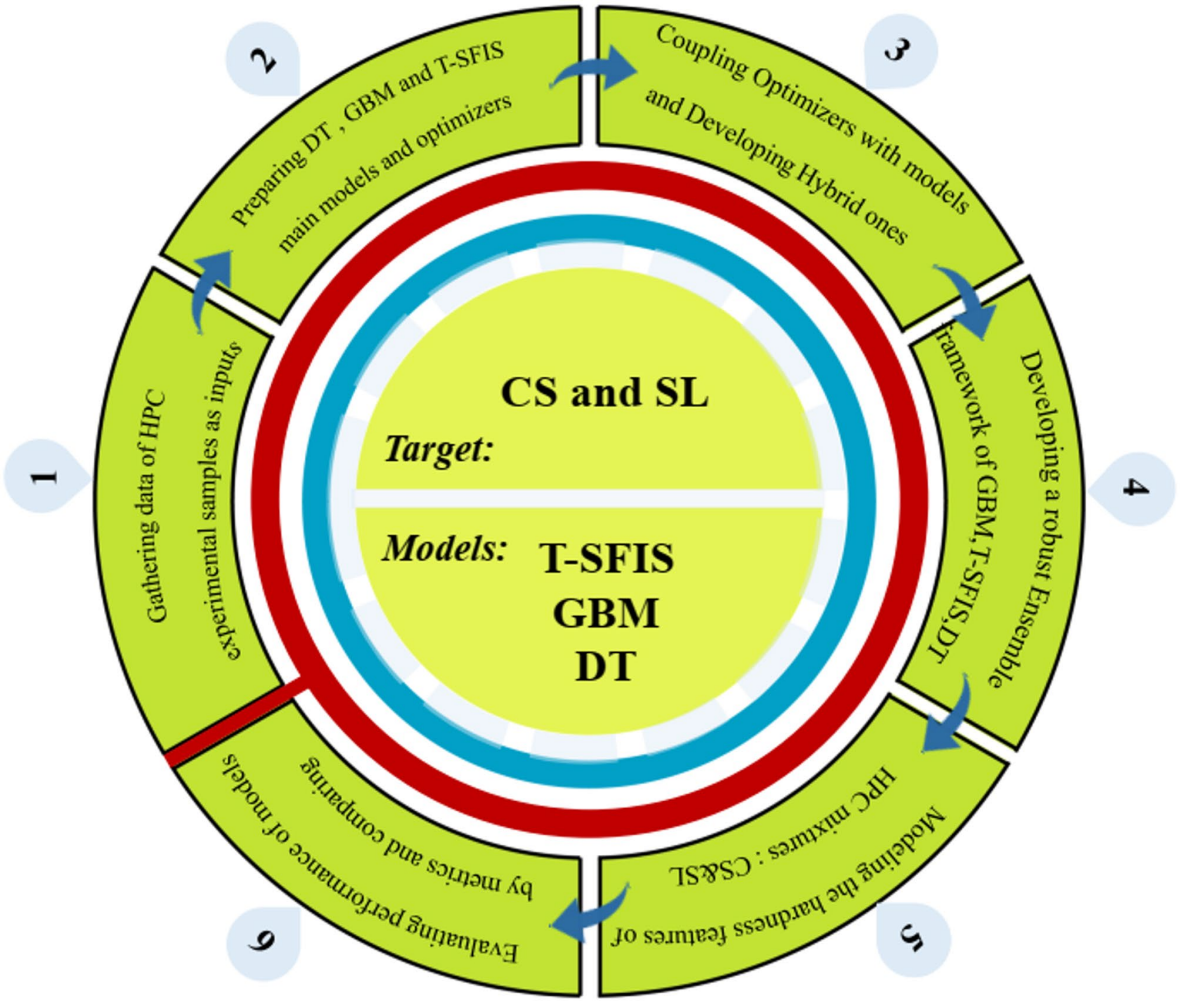
Phases taken into account for creating and assessing the ensemble and hybrid models.

This study’s originality is the creation of a novel hybrid and integrated framework that combines metaheuristic algorithms GWO and QPSO with T-SFIS, GBM, and DT models to forecast the strength characteristics of HPC with previously unheard-of precision. In contrast to earlier research that typically concentrates on single models or constrained optimization, this study outperformed state-of-the-art models like XGBoost R^2^ = 0.987, CatBoost R^2^ = 0.991, and deep learning R^2^ = 0.95 by combining metaheuristic optimization and combined approaches, achieving R^2^ = 0.998 for CS and 0.984 for flowability. Figure 1 illustrates the comprehensive phases of ongoing research aimed at stimulating the Strength characteristics of HPC mixtures. The main contributions of this research are as follows.

- This study employed advanced deep learning models such as T-SFIS, GBM, and DT, integrated with metaheuristic optimization algorithms like GWO and QPSO, to predict the Strength properties of HPC. These methods significantly improved the accuracy of predicting CS and slump flow rates, outperforming traditional approaches.
- Using SHAP-based sensitivity analysis and K-Fold cross-validation, the research identified the most influential factors affecting the Strength properties of HPC, such as the water-to-binder ratio and the use of additives. These methods enhanced the reliability of the models and ensured precise predictions.
- By integrating multiple machine learning models and optimizing them, the study introduced a new methodology for assessing the Strength properties of HPC. The results demonstrated that the GBQP hybrid model achieved the best performance for CS prediction, while the TSQP model was most effective for slump flow prediction.

The paper is organized as follows. Section “Materials and methodology” reports the Materials and Methodology. Section “Results and discussion” presents the Results and Discussion for the proposed approach as well as other existing methods. Finally, Section “Conclusion” discusses the conclusions and future work.

## Materials and methodology

### Data initialization

The dataset of 191 HPC mixtures, collected from^38^, required preprocessing to ensure data quality and consistency. Missing values, present in less than 5% of the entries (mainly for superplasticizer and fly ash), were imputed using median values to maintain statistical distributions, as the calculated mean can be skewed by outliers. Outliers, identified using the interquartile range (IQR) method (values beyond 1.5 × IQR), were identified in water-to-binder (W/B) and silica fume (SF) ratios for approximately 3% of the samples and were removed to avoid model bias. Inconsistencies, such as non-standard units for CS or slump flow, were reconciled by converting all measurements to MPa and mm, respectively, in accordance with ASTM standards. Feature standardization (zero mean, unity variance) was applied to normalize the input ranges and increased the convergence of the model during training (Table 1).

**Table 1:** Comparison of hybrid and combined models for predicting concrete properties.

Refs	Models	Optimization algorithms	Data	Predicted properties	Performance metrics	Limitations
^6^	Random Forest, XGBoost	Krill Swarm, Leopard Jaw	200 sustainable concrete mixes	CS	R^2^ = 0.98, RMSE = 2.1 MPa	High computational complexity
^7^	ANN, SVR	POA, WOA	150 UHPC mixes	CS, workability	R^2^ = 0.95–0.97, RMSE = 2.5–3.0 MPa	Limited data
^8^	XGBoost	CSA, WS, HH, FO	300 HPC mixes	CS	R^2^ = 0.987, RMSE = 2.3 MPa	Sensitive to data quality
^9^	ANN, SVR	GA, PSO	250 geopolymer mixes	CS	R^2^ = 0.96, RMSE = 2.8 MPa	Environmental conditions not considered
^10^	XGBoost	RS, GS, BO, PSO, GWO	200 recycled concrete mixes	Tensile strength	R^2^ = 0.97, RMSE = 0.5 MPa	Limited scalability
^14^	SVR	GA, PSO	180 HPC mixes	CS	R^2^ = 0.94, RMSE = 3.2 MPa	High computational intensity
^18^	ANN, Random Forest	Bagging, Boosting	220 HPC mixes	CS, workability	R^2^ = 0.93–0.96, RMSE = 3.5–4.0 MPa	Poor generalization to new data
^23^	ANN	GA	160 coal-ash concrete mixes	CS	R^2^ = 0.96, RMSE = 2.5 MPa	Workability not considered
This Work	GBM, DT, T-SFIS	GWO, QPSO	191 HPC mixes	CS, workability	R^2^ = 0.998 CS, 0.984 (slump), RMSE = 1.226 MPa CS, 3.233 mm (slump)	Dependence on experimental data

The assessment of slump Strength characteristics for high-performance concrete samples was conducted after 28 days. Additionally, a grain size of 7.2 and a specific density of 2.7 for coarse aggregates derived from crushed granite measuring 19 mm were utilized in the studies. Portland cement was chosen in accordance with ASTM standard Type I. Naphthalene-based superplasticizer was utilized to diminish water content and regulate the water-to-binder ratio. It is important to remember that aggregates consist of fine silica sand with a specific gravity of 2.61 and a fineness coefficient of 2.94. Table 2 shows the target values of the training section and the statistical characteristics of the input of the proposed model. The slump test conformed to ASTM C 143-90a standards following quick mixing in accordance with ASTM C 231-91b guidelines.

**Table 2:** The training section’s target values and model input’s statistical characteristics.

Variables	Unit	Category	Max	Min	Avg	SD
W/B	(%)	Input	47	19	31.14	8.73
W	(Kg/m^3^)	Input	182	139	152.23	13.02
S/A	(%)	Input	55	34	51.34	5.73
FA	(%)	Input	23	0	4.83	8.91
AE	(Kg/m^3^)	Input	0.098	0	0.042	0.05
SF	(%)	Input	27	0	6.32	8.83
SP	(Kg/m^3^)	Input	37.3	1.98	12.91	8.72
CS	(MPa)	Output	132	41	64.9	27.1
Slump	(mm)	Output	259	89	212.13	26.01

**Fig. 2:**
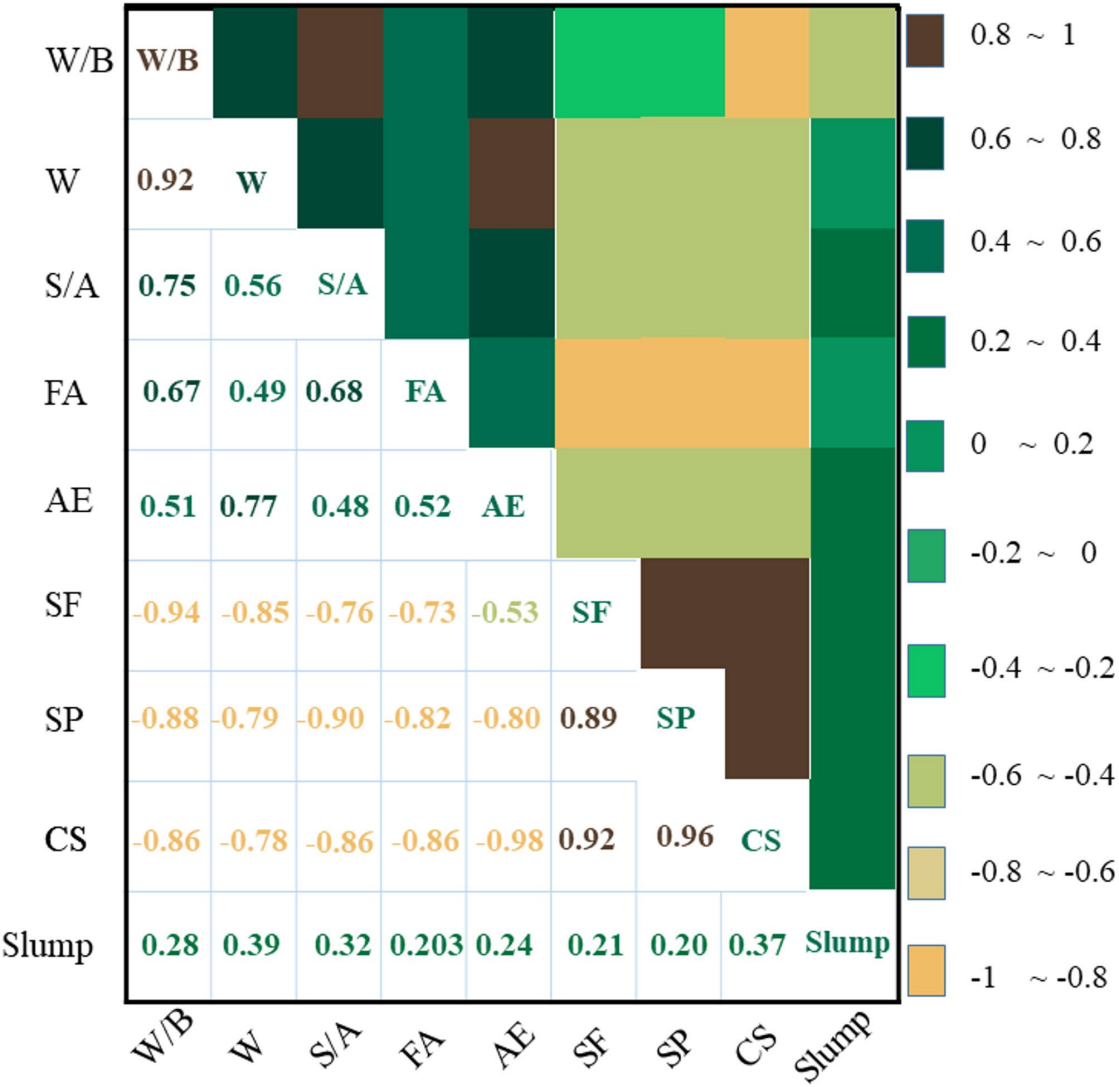
The values indicating the relationship between HPC components and Strength characteristics.

**Fig. 3:**
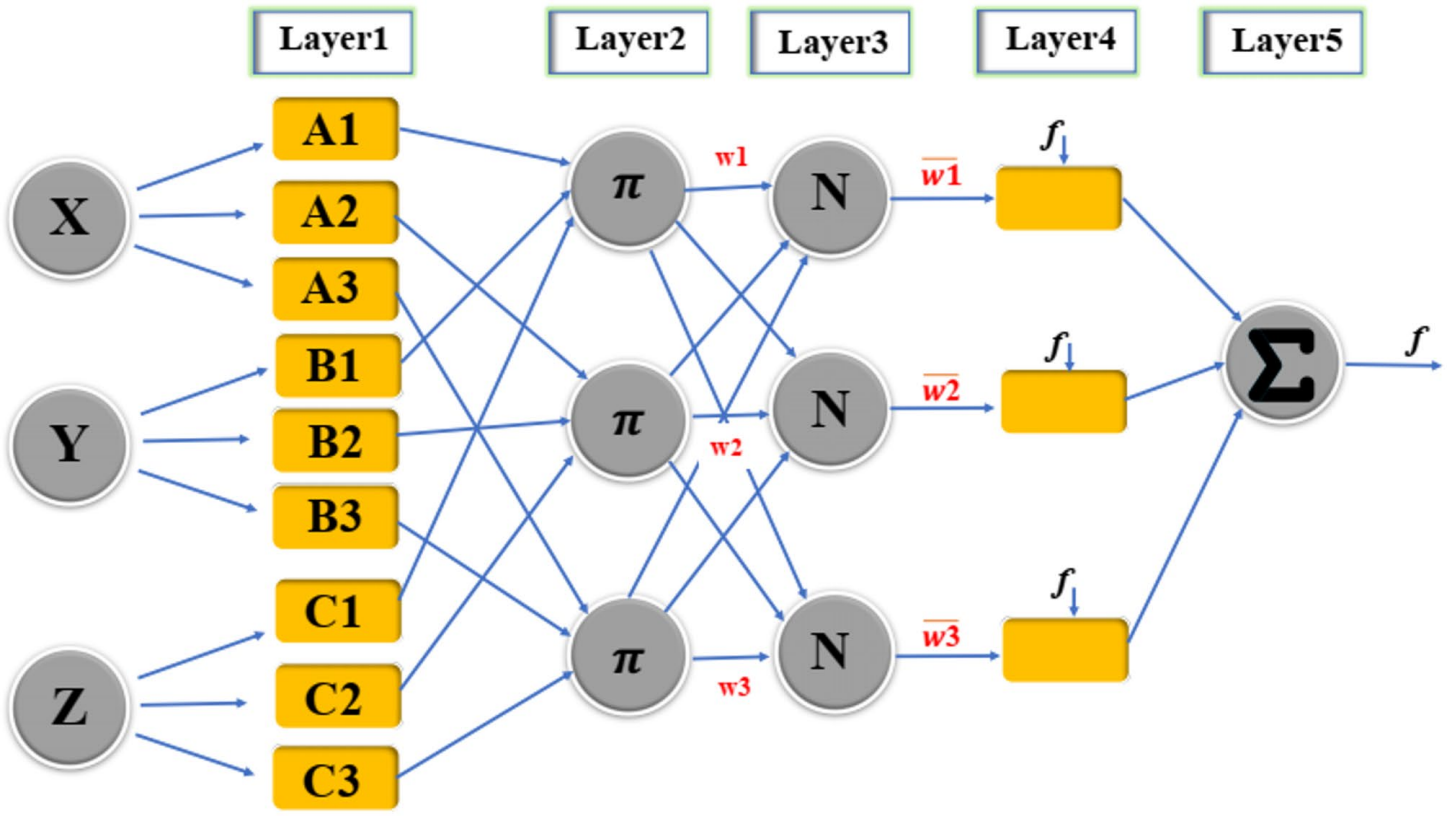
The structure of T-SFIS.

Figure 2 illustrates the relationships between the ingredients and the Strength characteristics of concrete samples. Furthermore, the heat maps in Fig. 3 illustrate the simultaneous impact of the water content variable (W) in conjunction with W/B, S/A, and SF on the slump and CS of HPC samples. The significant correlation coefficients between Strength parameters and HPC components are linked to silica fume and superplasticizer, exhibiting correlation values between 0.8 and 1 for CS and between 0.2 and 0.4 for slump.

### T-S fuzzy inference system (T-SFIS)

The T-SFIS network structure has two phases called the result and the hypothesis part. Training T-SFIS indicates specifying the parameters related to these phases. T-SFIS uses existing inputs to output data pairs during training. Then can obtain the IF–THEN fuzzy rules that connect these phases^40^. As shown in Fig. 3, the T-SFIS structure contains 5 layers. T-SFIS has a Two-input, one-output structure and 4 rules. Hierarchical structure of T-SFIS is defined below.

- Layer one

The first layer is referred to as the Fuzzification Layer. A fuzzification layer employs membership functions to derive fuzzy sets from input data. The parameters that define the format and membership functions are referred to as prerequisite parameters, with {a, b, c} representing the necessary parameter locations^41^. The membership degree is computed for each membership function by applying these parameters in Eqs. (1) and (2). The membership degrees acquired from this stratum are represented by ${\delta }_{x}$ and ${\delta }_{y}$:

1 $${\delta }_{{A}_{i}}\left(x\right)=gbellmf\left(x;a,b,c\right)=\frac{1}{1+{\left|\frac{x-c}{a}\right|}^{2b}}$$

2 $${U}_{i}^{1}={\delta }_{{A}_{i}}\left(x\right)$$

b) Layer two

The dominant layer is referred to as layer two. The fuzzy layer employs the computed membership values to provide rule trigger intensities (vi)^42^. Reinforcement learning approaches for nonlinear systems^42^ complement metaheuristic algorithms like GWO and QPSO in optimizing HPC prediction models. The vi value is calculated by multiplying the membership values as indicated in Eq. (3):

3 $${U}_{i}^{2}={v}_{i}={\delta }_{{A}_{i}}\left(x\right).{\delta }_{{B}_{i}}\left(y\right) i=\text{1,2}.$$

c) Layer three

The normalizing level designates layer three. Determine the normalized firepower corresponding to each rule. Moreover, the normalized value represents the ratio of the fire intensity of the *ith* rule to the total sum of all fire intensities, expressed as:

4 $${U}_{i}^{3}={w}_{i}=\frac{{v}_{i}}{{v}_{1}+{v}_{2}+{v}_{3}+{v}_{4}} i\in \{1, 2, 3, 4\}$$

d) Layer four

Defuzzification Layer is the designation for layer four. Fuzzy-based control methods, such as those for multi-agent systems^43^, support the application of T-SFIS in modeling HPC properties. A weighted rule value is computed for each node in this layer. This value is ascertained utilizing a first-order polynomial in Eq. (5):

5 $${U}_{i}^{4}={w}_{i}{f}_{i}={w}_{i}({q}_{i}x+{p}_{i}y+{r}_{i})$$

where {${q}_{i}$, ${p}_{i}$, ${r}_{i}$} denotes the parameter set and ${w}_{i}$ denotes the normalization layer output. The consequence parameter is their name. The number of follow-up parameters for each rule is one more than the number of inputs.

e) Layer five

The supplementary layer is referred to as its name. T–S fuzzy models, applied in secure control^44^, provide a robust framework for predicting HPC strength properties. T–S fuzzy-based control for complex systems^48^ supports the use of T-SFIS in modeling HPC properties. The defuzzification layer produces the real result of T-SFIS by aggregating the outputs derived from each rule as presented in Eq. (6)^44^.

6 $${U}_{i}^{5}=\sum_{i}^{n}{w}_{i}{f}_{i}=\frac{\sum_{i}^{n}{v}_{i}{f}_{i}}{\sum_{i}^{n}{v}_{i}}$$

### Gradient boosting (GBM)

GBM can be utilized for both regression and classification tasks. In the context of a general regression issue, the training dataset R can be expressed as shown in Eq. (7):

7 $$D=\left\{\left({X}_{1},{Y}_{1}\right),\left({X}_{2},{Y}_{2}\right),\dots ,\left({X}_{n},{Y}_{n}\right)\right\}$$

$\left({X}_{i},{Y}_{i}\right)(i=\text{1,2},\dots ,n)$ denotes the training data set for the ith sample, where n determines the total number of samples. Xi represents the input data vector and Yi represents the output data values. This can be employed to train a primary (or weak) learner $W\left(X\right)$ with a particular learning algorithm^45^. The relative estimation error ${e}_{i}$ for each sample is represented in Eq. (8):

8 $${e}_{i}=F({Y}_{i}.W{(X}_{i}))$$

in Eq. (8), F denotes the loss function and has three options including square decay, linear decay, and exponential decay. A simple linear loss function is employed in Eq. (9):

9 $${e}_{i}=\frac{\left|{Y}_{i}-W{(X}_{i})\right|}{A}$$

Here $A=max\left|{Y}_{i}-W{(X}_{i})\right|$ denotes the maximum absolute estimation error for all samples.

The purpose of GBMBoost is clearly to generate a sequence of weak learners ${W}_{c}(X)$ one after the other. This is because there is only one weak learner that performs very poorly. $c=\text{1,2},\dots ,N$, and combine them to construct $W{(X}_{i})$ by a good learner through combinatorial methods. Regression issues are combinatorial methods:

10 $$W\left(X\right)=\text{h}\sum_{c=1}^{N}\left(ln\frac{1}{{x}_{c}}\right)w(X)$$

In Eq. (10), ${x}_{c}$ determines the weights of the weak learner ${W}_{c}(X)$ and $w(X)$ denotes the median of all ${x}_{c}{W}_{c}(X)$, $h\upepsilon (\text{0,1})$ is the regularization factor used to avoid overfitting issues.

Weak learners ${W}_{c}(X)$ and their weights ${x}_{c}$ are developed using the original training data’s modified version. This is done by a so-called rebalancing technique^50,51^. This is obtained by changing each sample’s distribution weight according to the estimation error of the recent weak learner ${W}_{c-1}(X)$. Misestimated samples are weighted more heavily and thus represented more attention to the following training process. This is an iterative method. At $c=\text{1,2},\dots ,N$ iterations, the weak learner gets ${W}_{c}(X)$ and the relative estimation error ${e}_{ci}$ is determined according to Eq. (9). So, the total error rate ${r}_{c}$ for this step is:

11 $${e}_{c}=\sum_{i=1}^{n}{e}_{ci}$$

Then the weight of weak learners represented in Eq. (12)

12 $${x}_{c}=\frac{{e}_{c}}{1-{e}_{c}}$$

In addition, the distribution’s weight ${v}_{c+1,i}$ for each sample in the next step of training represented in Eq. (13):

13 $${v}_{c+1,i}=\frac{{v}_{c,i}\times {x}_{c}^{1-{e}_{ci} }}{\sum_{i=1}^{n}{v}_{c,i}\times {x}_{c}^{1-{e}_{ci}}}$$

**Fig. 4:**
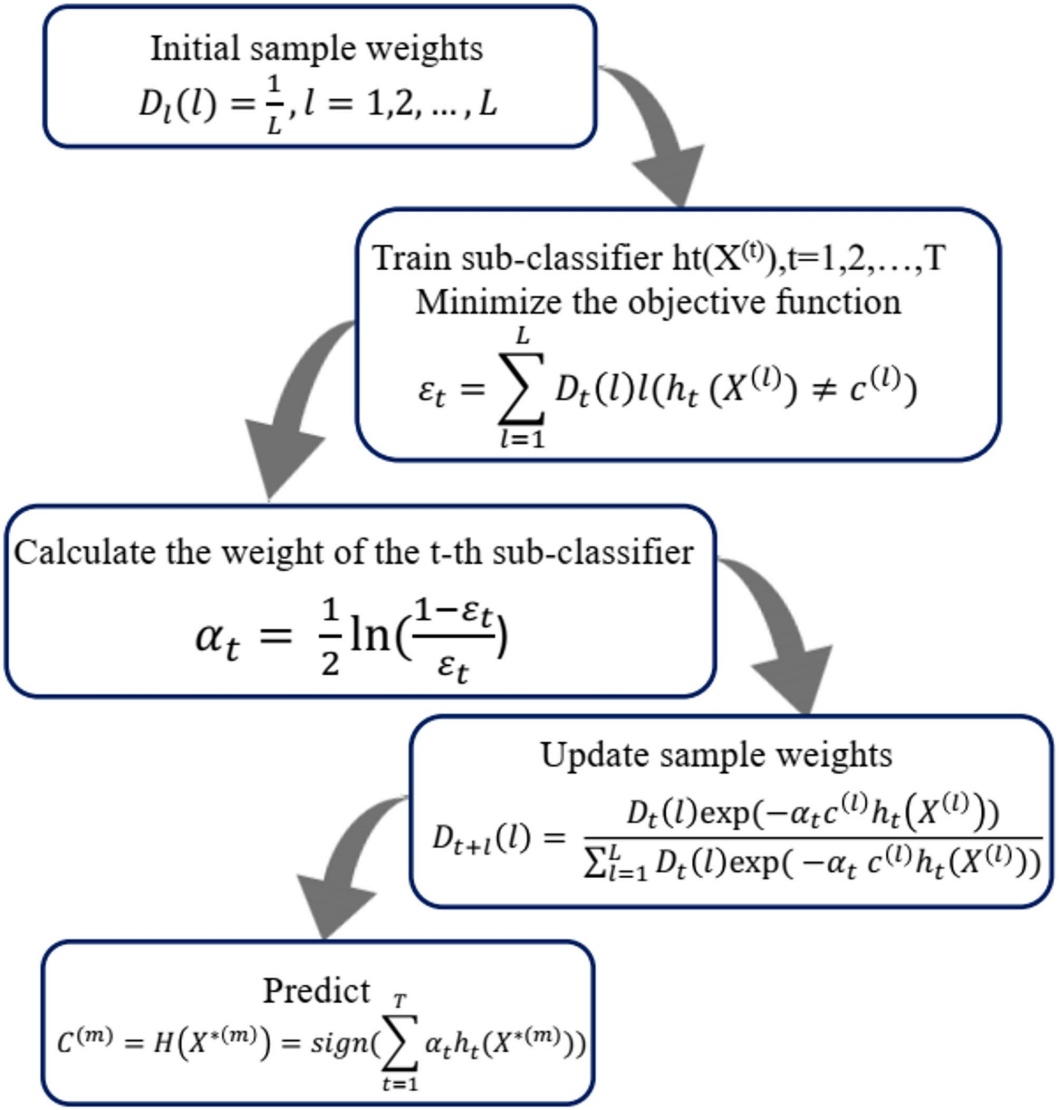
The flowchart of GBM boosting model.

In Eq. (13), ${v}_{c,i}$ and ${x}_{c}$ are two weights. ${v}_{c,i}$ indicates the importance of the training data samples used to increase the misestimated samples’ weight to indicate that the next step will learn to be acceptable. ${x}_{c}$ is used to determine weak learners and determine that more accurate weak learners have a higher impact on the final output. Figure 4 has exhibited the flowchart of Gradient Boosting Machines modeling processes.

Based on their exceptional qualities and effective uses in tangible property prediction, GBM, DT, and T-SFIS were chosen as the foundation models for creating hybrid and combination frameworks in this study. Because of its gradual boosting technique and capacity to capture intricate nonlinear interactions between concrete components, including silica fume, water-to-binder ratio, and strength qualities (CS and flowability), GBM is ideally suited for regression problems^30^. With metaheuristic optimization (GWO and QPSO), this model’s high degree of hyperparameter tuning flexibility is further improved. Because of its ease of use, interpretability, and capacity to minimize mistakes in integrated frameworks (such bagging and boosting), DT was chosen. It has demonstrated performance in real prediction models like^31^. T-SFIS, which blends fuzzy logic with neural network learning, has been effectively applied to forecast concrete qualities in earlier research like^29^ and is perfect for modeling uncertainties and nonlinear interactions in HPC mixes. These models were chosen to offer a strong foundation for precise HPC property prediction because of their complimentary characteristics (accuracy of GBM, interpretability of DT, and uncertainty management of T-SFIS).

### Decision tree (DT)

DT are a widely utilized approach and system for developing classifiers in data mining. Classification algorithms can analyze substantial volumes of data in data mining^40^. Adaptive control techniques for nonlinear systems, such as backstepping^41^, enhance the robustness of models like T-SFIS for HPC property prediction. This document focuses on DT algorithms within the broader category of machine learning classification methods.

Entropy is utilized to quantify the impurity of a dataset or to assess unpredictability. Entropy values consistently range from 0 to 1. A value of zero can be both appropriate and inappropriate. A closure of 0 is more appropriate. The classification entropy of set D for the d-state can be ascertained if the objects possess distinct features:

14 $$D=\sum_{i=1}^{d}{P}_{i}.log{2}^{{P}_{i}}$$

In Eq. (14), ${P}_{i}$ denotes the ratio of the number of samples in the subset to the ith property value.

The acquired information serves as the metric for segmentation and is referred to as mutual information. The intuition reveals the extent of knowledge regarding the values of the random components^53^. Conversely, the superior and elevated the value. The data gain (D, F) is delineated as per the entropy specification provided in Eq. (15):

15 $$(D,F)=\sum_{w\in W(F)}^{d}\frac{\left|{S}_{w}\right|}{\left|S\right|}.({S}_{w})$$

**Fig. 5:**
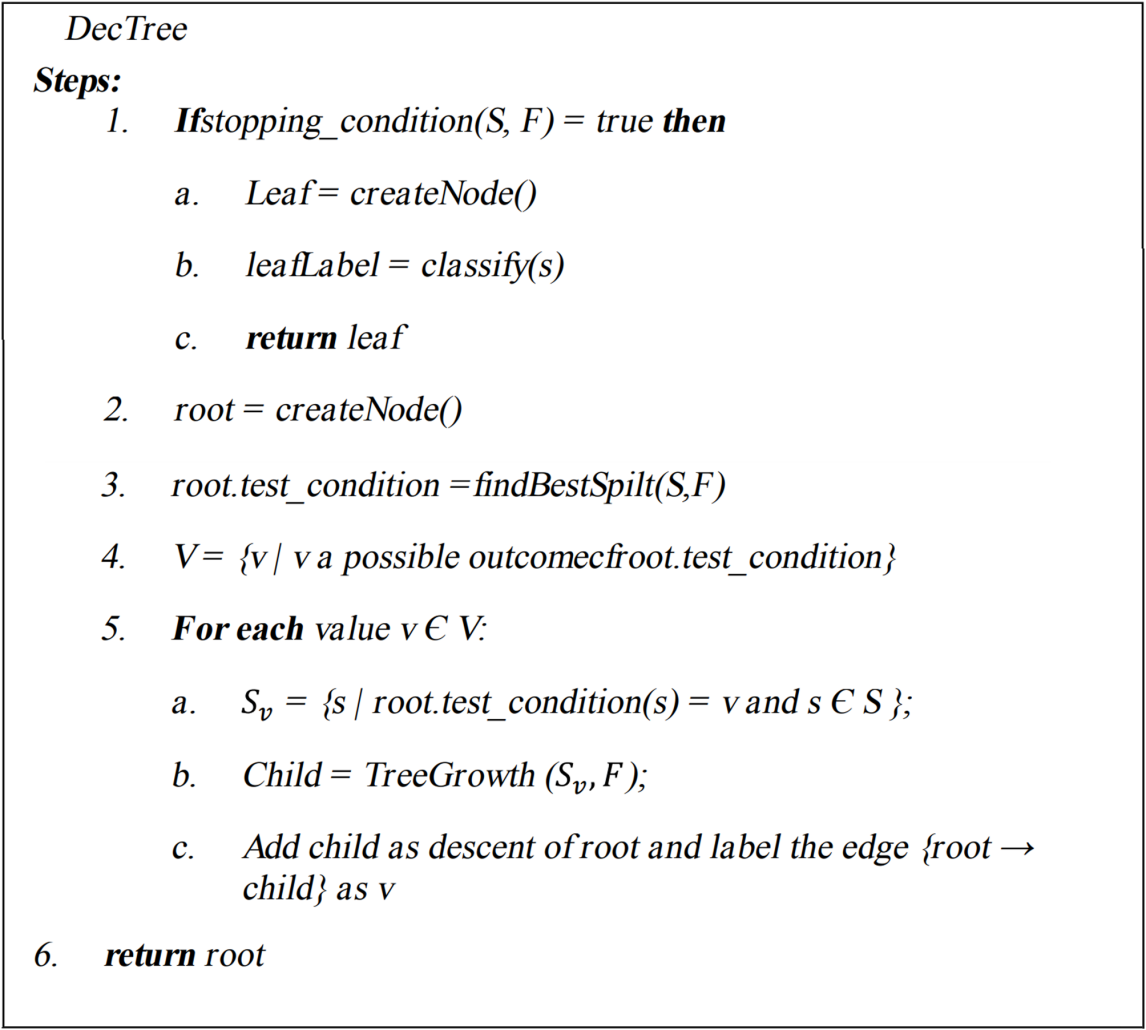
The pseudo code of DT approach.

**Fig. 6:**
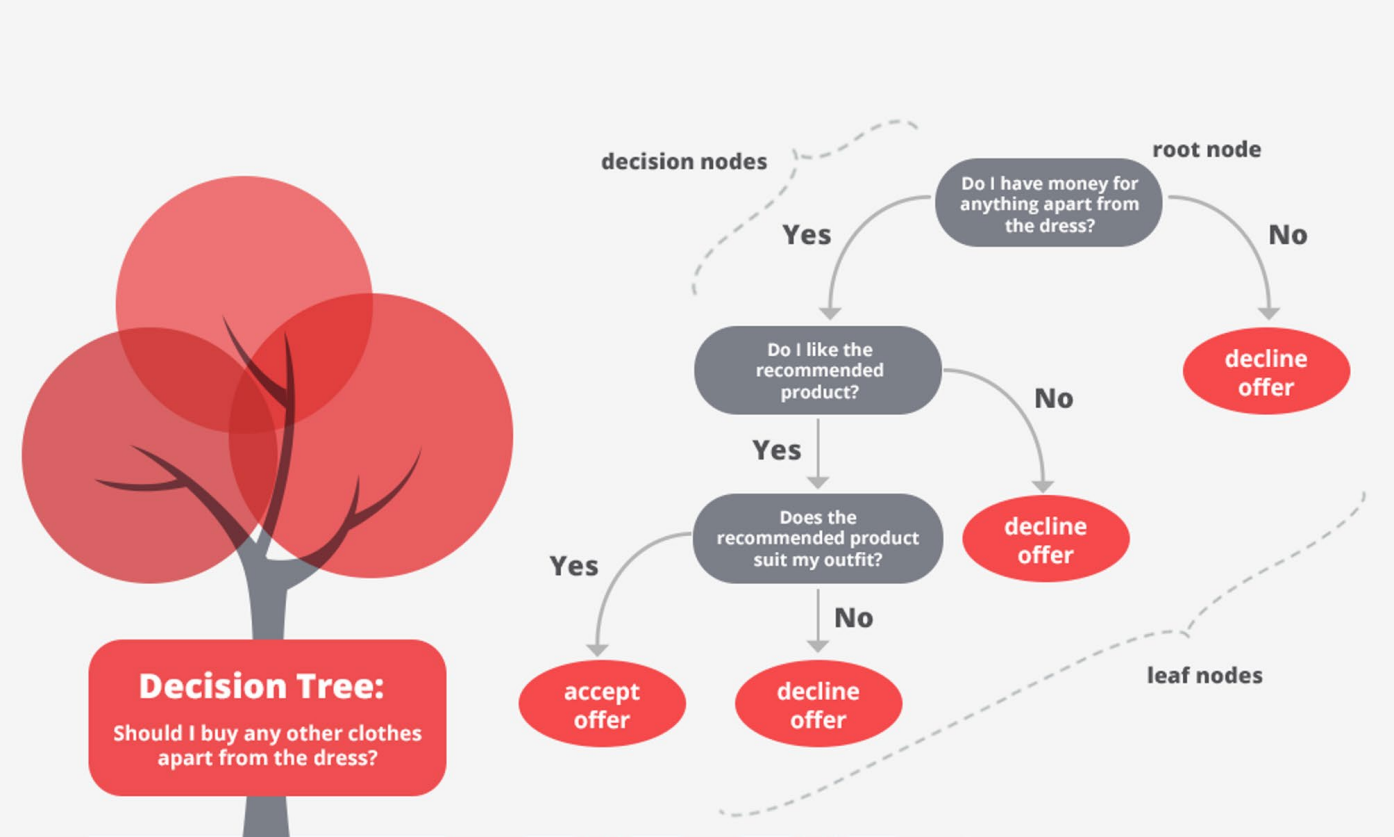
The structure of DT approach.

In Eq. (15), $W\left(F\right)$ denotes the range of the feature F, whereas ${S}_{w}$ represents the subset S corresponding to the feature value of w^42^. Figures 5 and 6 illustrate the pseudocode and framework of the DT methodology^43^.

### Grey wolf optimizer (GWO)

The GWO algorithm was formulated based on the ideas of Grey theory, as well as the fundamental concepts of fractals and chaos. The GWO technique utilizes a set of points that denote several optimal positions within the Sierpinski triangle, evaluating candidate solutions (X) while sustaining the population of solutions using numerous natural evolutionary algorithms that are enhanced by stochastic modifications and exploration. The candidate solution (*Xi*) comprises many decision parameters $\left({x}_{i,j}\right)$ that denote the positions of eligible points within the Sierpinski triangle in the context of GWO. The Sierpinski triangle serves as a search space in GWO for potential solutions. The mathematical representation of the Sierpinski triangle is articulated as demonstrated in Eq. (16)^35^:

16 $$X=\left[\begin{array}{c}{X}_{1}\\ {X}_{2}\\ \vdots \\ {X}_{n}\end{array}\right]=\left[\begin{array}{c}{x}_{1}^{1}\\ {x}_{2}^{1}\\ \cdots \\ {x}_{n}^{1}\end{array} \begin{array}{c}{x}_{1}^{2}\\ {x}_{2}^{2}\\ \cdots \\ {x}_{n}^{2}\end{array} \begin{array}{c}\cdots \\ \cdots \\ \ddots \\ \cdots \end{array} \begin{array}{c}{x}_{1}^{d}\\ {x}_{2}^{d}\\ \vdots \\ {x}_{n}^{d}\end{array}\right]$$

where in *n* is the possible number of points and *d* the points’ dimensions. Finding suitable points’ locations should be done in the exploring area accidently that is defined in Eq. (17):

17 $${x}_{i}^{j}\left(0\right)={x}_{i,min}^{j}+rand\times \left({x}_{i,max}^{j}-{x}_{i,min}^{j}\right), \left\{\begin{array}{c}i=\text{1,2},\dots ,n.\\ j=\text{1,2},\dots ,d.\end{array}\right.$$

The initial locations of possible points are indicated by ${x}_{i}^{j}\left(0\right)$. The upper and lower tolerances of the solution for choice variable j are denoted as ${x}_{i,max}^{j}$ and ${x}_{i,min}^{j}$ respectively.

The variable *rand* is a random value inside the interval [0 to 1]. A temporary triangle is established in the Global Best (*GB*) position to identify the initial suitable point inside the search area (*Xi*). Consequently, the Global Best positions are utilized for the ideal solutions of applicants exhibiting a high level of fitness identified. The initial accidental point, which is assessed with equal probability to the first eligible point (Xi), is presently regarded as^44^.

Utilizing the grey wolf algorithm to regulate some parameters necessitating limitations for the seeds traversing the search space, various randomly generated variables are employed in the mathematical expression presented in Eq. (18):

18 $${S}_{i}^{j}={X}_{i}+{a}_{i}\times \left({b}_{i}\times GB-{c}_{i}\times {MG}_{i}\right), i=\text{1,2},\dots ,n.$$

**Fig. 7:**
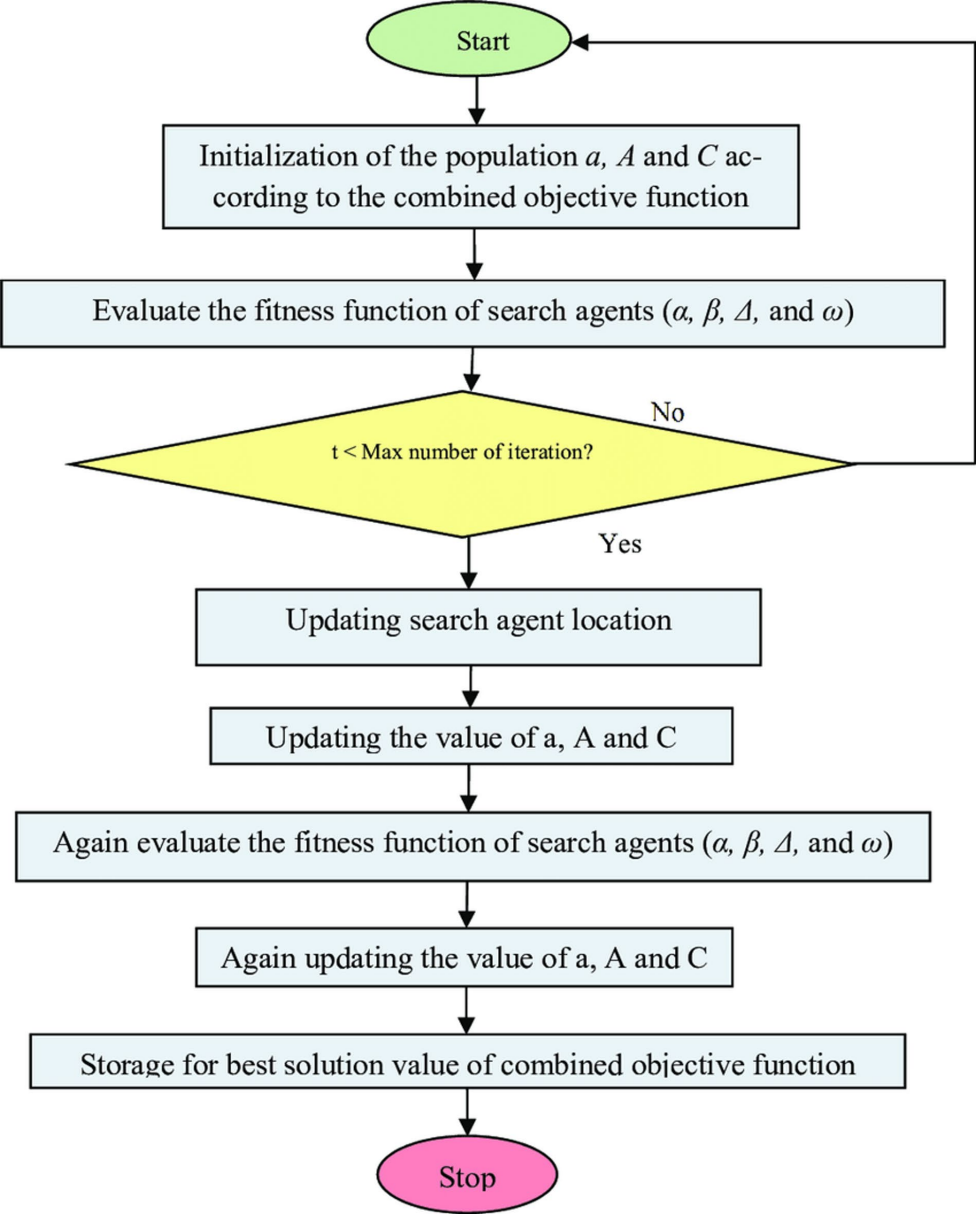
The flowchart of Grey Wolf Optimizer.

Here, *Xi* represents the ith solution candidate, a random value of ${a}_{i}$, with integer values of 0 or 1, is utilized to imitate the movement of *GB* limits, and *MGi* denotes the mean of the acceptable point value for the three vertices in the temporal triangle. Four distinct equations were utilized to define limits for optimizing the calculation process, employing the established GWO algorithm to allocate ${a}_{i}$ for modeling the constraints. The four limitations are inadvertently employed in Eq. (19)^53^.

19 $${a}_{i}=\left\{\begin{array}{c}Rand\\ 2\times Rand\\ \left(\mu \times Rand\right)+1\\ \left(\theta \times Rand\right)+(\sim \theta )\end{array}\right.$$

where *Rand* is a random number uniformly distributed between 0 and 1, and $\mu$, $\theta$ are random integer parameters^35^. Figure 7 illustrates the flowchart of the GWO.

### Quantum-behaved QPSO

The control parameters of the PSO must delineate diverse strategies within the optimization process to enhance convergence and efficiency. QPSO utilizes an appropriate and effective cursor angle function to formulate PSO control parameters for target attainment. Each particle is assigned a function of these phase angles, utilizing scalar phase angles such as cosine and sine to represent the PSO control parameters for the development of various approaches in QPSO. The ith particle is represented as ${{\overrightarrow{X}}_{i}, \delta }_{i}$ , characterized by the pointer angle ${\delta }_{i}$ and the magnitude vector ${\overrightarrow{X}}_{i}$.

In QPSO, the inertial weight is assigned a value of zero; however, this methodology can be advanced by incorporating concepts from other improved PSOs. The proposed particle movement model in QPSO is defined as^37^:

20 $${W}_{i}^{Iter}=f\left({\delta }_{i}^{Iter}\right)\times \left({Fbest}_{i}^{Iter}-{X}_{i}^{Iter}\right)+u({\delta }_{i}^{Iter})\times ({Ubest}^{Iter}-{X}_{i}^{Iter})$$

Through studying some functions $f\left({\delta }_{i}^{Iter}\right)$ and $u({\delta }_{i}^{Iter})$, the functions selected for QPSO are in Eqs. (21) and (22):

21 $$f\left({\delta }_{i}^{Iter}\right)={\left|{cos\delta }_{i}^{Iter}\right|}^{2\times {sin\delta }_{i}^{Iter}}$$

22 $$u\left({\delta }_{i}^{Iter}\right)={\left|{sin\delta }_{i}^{Iter}\right|}^{2\times {cos\delta }_{i}^{Iter}}$$

The proposed functions can provide enhancement, decrement, equalization, inversion of value, and amplification. It is merely the function of the particle’s pointing angle. Attitudes render locally desired, exploratory characteristics adaptable and globally harmonious. QPSO is a highly effective non-parametric adaptive technique for circumventing local optimizations and preventing premature convergence. The PSO algorithm has a general disadvantage.

23 $${W}_{i}^{Iter}={\left|{cos\delta }_{i}^{Iter}\right|}^{2\times {sin\delta }_{i}^{Iter}}\times \left({Fbest}_{i}^{Iter}-{X}_{i}^{Iter}\right)+{\left|{sin\delta }_{i}^{Iter}\right|}^{2\times {cos\delta }_{i}^{Iter}}\times ({Ubest}^{Iter}-{X}_{i}^{Iter})$$

Then update the particle position using the following formula:

24 $${\overrightarrow{X}}_{i}^{\text{Iter}+1}={\overrightarrow{X}}_{i}^{Iter}+{\overrightarrow{W}}_{i}^{Iter}$$

Subsequently, ($Ubest$) and ($Fbest$) are established as the global best and personal best, respectively, analogous to the PSO method. The maximum velocity and the particle’s phasor angle are revised as follows:

25 $${\overrightarrow{\delta }}_{i}^{\text{Iter}+1}={\updelta }_{i}^{\text{Iter}}+Y\left(\delta \right)\times \left(2\pi \right)={\updelta }_{i}^{\text{Iter}}+\left|{\text{sin}(\delta }_{i}^{Iter})+{\text{cos}(\delta }_{i}^{Iter})\times \left(2\pi \right)\right|$$

26 $${W}_{i,max}^{Iter+1}=W\left(\delta \right)\times \left({X}_{max}-{X}_{min}\right)={\left|{cos\delta }_{i}^{Iter}\right|}^{2}\times \left({X}_{max}-{X}_{min}\right)$$

**Fig. 8:**
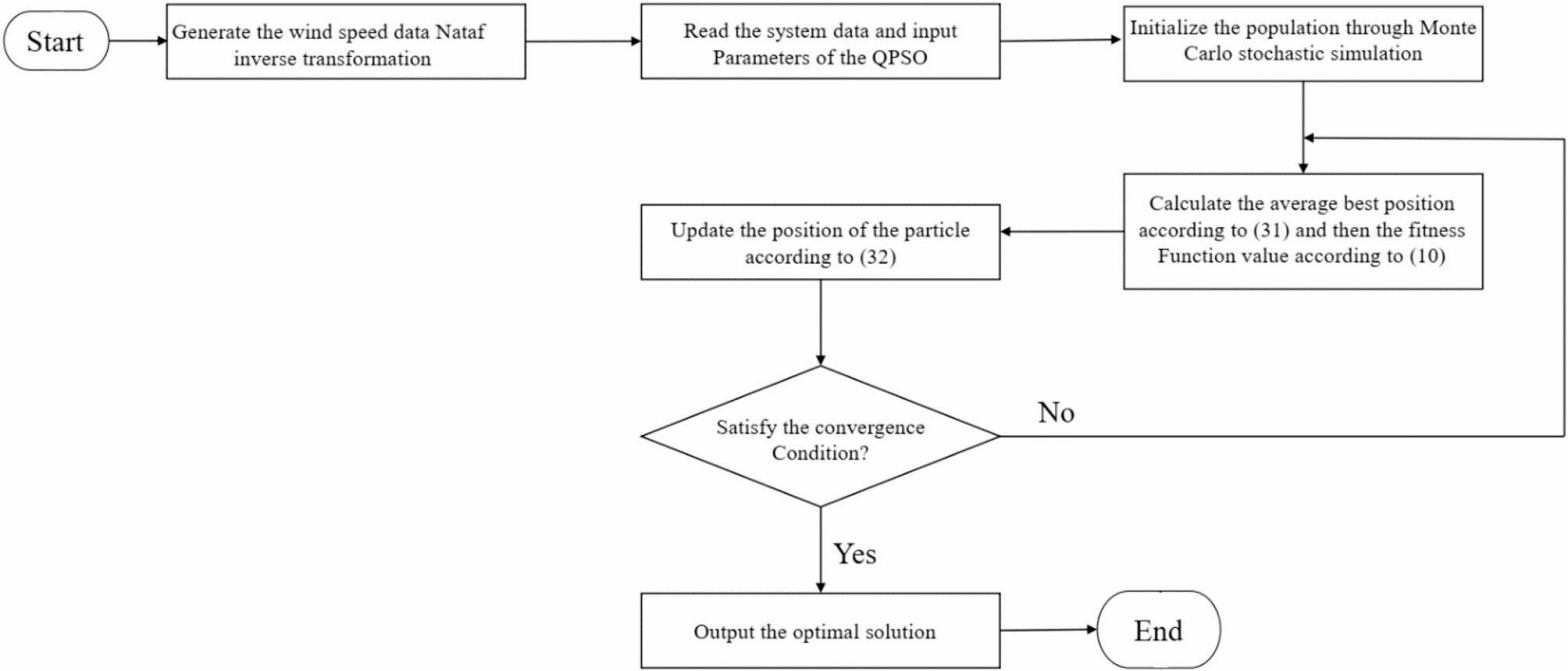
The flowchart of Quantum-behaved Particle Swarm Optimization.

The overall mechanism of working QPSO has been indicated in flowchart of Fig. 8.

Based on their exceptional qualities and prior achievements in related issues, the metaheuristic algorithms Grey Wolf Optimizer (GWO) and Quantum-behaved Particle Swarm Optimization (QPSO) were chosen to optimize the T-SFIS, GBM, and DT models. With just a few control settings, GWO is easy to implement and offers a good balance between exploration and exploitation. It was inspired by the hunting behavior of gray wolves. When it comes to forecasting the tensile strength of recycled concrete^10^ and solving routing issues^50^, this method has demonstrated excellent performance in optimizing predictive models in civil engineering. However, QPSO, a more sophisticated form of PSO, uses quantum mechanical concepts to provide faster convergence and better avoidance of local optima, making it appropriate for high-dimensional problems with intricate nonlinear relationships, like steel structure failure^37^ and concrete property prediction^40^. In contrast to more conventional techniques like grid search, these two algorithms were chosen for the current study due to their capacity to optimize the hyperparameters of machine learning models and enhance prediction accuracy.

### Developing ensemble and hybrid models

Three distinct prediction models T-SFIS, GBM, and DT were integrated with metaheuristic optimization algorithms, GWO and QPSO, in three configurations to establish a novel methodology for evaluating the efficacy of the developed frameworks: i) individual mode (T-SFIS, GBM, DT), ii) hybrid mode (T-SFIS + QPSO = TSQP, GBM + QPSO = GBQP, DT + QPSO = DTQP, T-SFIS + GWO = TSGW, GBM + GWO = GBGW, DT + GWO = DTGW), and iii) ensemble mode (DT + GBM + T-SFIS = DGT). The primary objective of this research is to construct hybrid frameworks by integrating the principal prediction models of T-SFIS, GBMBoost, and DT with two optimization methods, hence introducing a novel methodology for tuning the estimation models. Optimizers endeavor to modify the programming procedures of models throughout the modeling phase. Some arbitrary parameters exist in the algorithms of models to which they are optimized. Consequently, by identifying the causal state variables of models at optimal levels, the models can operate at peak performance and accurately simulate the Strength features of HPC mixtures.

**Fig. 9:**
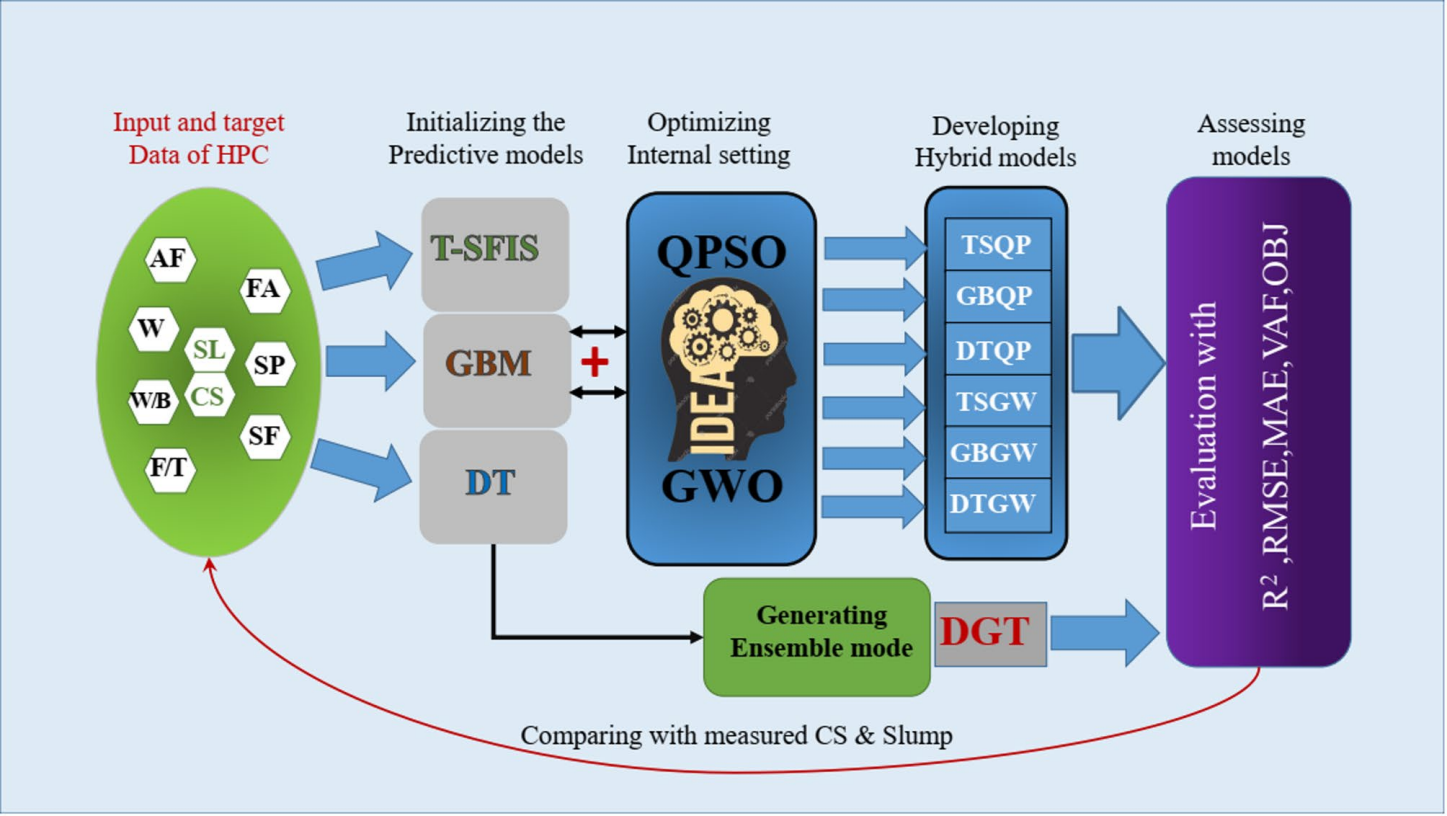
An overview of the hybrid and ensemble methodologies used to describe the Strength properties of HPC mixtures.

Conversely, ensemble methods are learning algorithms that integrate numerous distinct machine learning (ML) models to improve predictive performance^45^. The outcome is a conclusive model that surpasses the separate models. Ensemble approaches encompass bagging and boosting. Bagging seeks to enhance classification by amalgamating predictions from independently trained models within a randomly generated training set. The bagging method involves constructing multiple independent predictors and calculating the average of the estimators. It provides a predictor with reduced variance in comparison to independent predictors. Boosting is an ensemble meta-algorithm that amalgamates a collection of weak classifiers to construct a robust classifier. Incrementally construct the ensemble by continuously training new models and emphasizing misclassified training instances from prior models. The estimators are developed successively to minimize the bias of the final aggregated estimator. Figure 9 illustrates the schematic representation of the development of hybrid and ensemble models.

### K-Fold cross-validation and sensitivity analysis

**Fig. 10:**
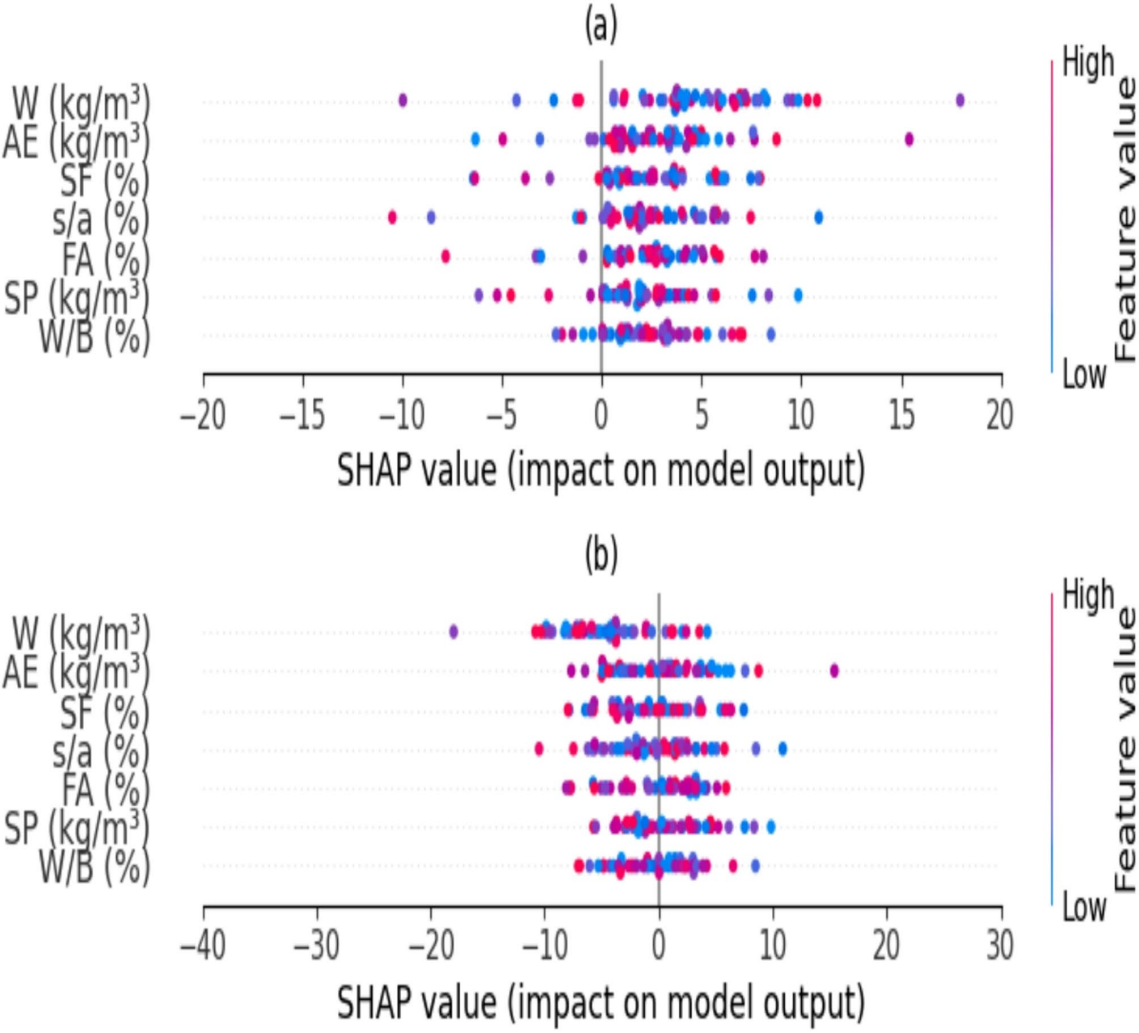
Sensitivity analysis for modeling (**a**) CS and (**b**) Slump.

K-fold cross-validation is frequently employed to mitigate the random sampling bias linked to training and to maintain data samples. Kohavi’s evaluation indicates that the 10 × validation method offers dependable variance with low computational time^46,47^. The model’s performance in classifying the fixed dataset into 10 subsets is evaluated using a tenfold cross-validation technique. For testing reasons, he selects a certain portion of the data, while utilizing the remaining subset in each of the 10 rotations for model building and validation to train the chosen model. The test subset is analyzed to verify the algorithm’s accuracy. The algorithm’s accuracy is expressed as the mean accuracy derived from 10 models throughout 10 validation iterations. The sensitivity analysis of inputs is acknowledged to diminish the cost function throughout the modeling phase and mitigate biases that arise in computational stages. Consequently, the SHapley Additive exPlanations (SHAP) methodology^48,49^ was employed. Figure 10 illustrates the SHAP sensitivity analysis for the current research modeling work. According to plot (a) in Fig. 10, the superplasticizer and the water to binder ratio (W/B) are the primary factors influencing CS, significantly altering CS values more than other variables. In Fig. 10 plot (b), the parameters of the fine to total aggregates ratio (s/a), fly ash, and superplasticizer are among the most influential factors affecting the slump flow values in the slump test.

### Predictive performance evaluation indicators

To improve the predicted accuracy of the Gradient Boosting Machine (GBM) for HPC strength parameters, hybrid models GBQP and TSQP were created. Because of the intricacy of HPC mix design and the nonlinear interactions between inputs (such as silica fume and water-to-binder ratio), additional optimization is required beyond the grid search of regular GBM. By combining GBM with metaheuristic methods, such as Quantum-behaved QPSO for TSQP and GWO for GBQP, hybridization improves model fit to the 191 HPC dataset by optimizing hyperparameters. Hybridization is required since it can overcome GBM’s shortcomings in identifying complex patterns. GBQP attained R^2^ = 0.998, RMSE = 1.226 MPa for CS, and TSQP recorded R^2^ = 0.984, RMSE = 3.233 mm for slump flow, which represents 8–10% improvements above normal GBM (R^2^ = 0.92, RMSE = 3.45 MPa for CS; R^2^ = 0.90, RMSE = 5.67 mm for slump flow).

The subsequent indicators are employed to assess the efficacy of the pertinent models. The indicators comprised the mean absolute error (MAE), coefficient of determination (R^2^), root mean squared error (RMSE), variance account factor (VAF), and objective detection metric (OBJ), calculable by Eqs. (27)–(31):

27 $${R}^{2}={(\frac{{\sum }_{d=1}^{D}\left({n}_{d}-\overline{n }\right)\left({c}_{d}-\overline{c }\right)}{\sqrt{\left[{\sum }_{d=1}^{D}{\left({n}_{P}-n\right)}^{2}\right]\left[{\sum }_{d=1}^{D}{\left({c}_{d}-\overline{c }\right)}^{2}\right]}})}^{2}$$

28 $$RMSE=\sqrt{\frac{1}{D}{\sum }_{d=1}^{D}{\left({c}_{d}-{n}_{d}\right)}^{2}}$$

29 $$MAE=\frac{1}{D}\sum_{d=1}^{D}\left|{c}_{d}-{n}_{d}\right|$$

30 $$VAF=\left(1-\frac{var({n}_{d}-{c}_{d})}{var({n}_{d})}\right)\times 100$$

31 $$OBJ=\left(\frac{{n}_{train}-{n}_{test}}{{cn}_{train}+{n}_{test}}\right)\frac{{ MAE}_{test}+{RMSE}_{train}}{1+{R}_{train}^{2}}+\left(\frac{{2n}_{train}}{{n}_{test}+{n}_{train}}\right)\frac{{RMSE}_{test}-{MAE}_{test}}{1+{R}_{test}^{2}}$$

where ${n}_{d}$ determines the measured value, $\overline{n }$ is the mean of the measured value, d shows the samples’ number, ${c}_{d}$ indicates the predicted value, $\overline{c }$ determines the mean of the predicted values, and $D$ is the data set’s number.

Because of its strong performance in regression tasks, especially when it comes to modeling nonlinearT1 complicated nonlinear interactions in the design of HPC mixes, the GBM was chosen as the foundational framework for the GBQP and TSQP models. GBM’s iterative boosting method, which adds weak learners one after the other to minimize a loss function, is ideal for capturing the complex relationships between Strength parameters (CS and slump flow) and HPC constituents (such as fly ash, silica fume, and water-to-binder ratio). Furthermore, GBM’s adaptability in hyperparameter tuning allows it to work with metaheuristic optimization methods such as Quantum-behaved QPSO and GWO, allowing for the creation of extremely accurate hybrid models (GBQP and TSQP). GBM is a perfect fit for this study since it provides a mix between interpretability and performance when compared to other sophisticated models like XGBoost and CatBoost.

For a relatively small dataset of 191 HPC mixtures, hybrid models integrating T-SFIS, GBM, and DT with GWO and QPSO were chosen because, even with limited data, they were able to capture complex nonlinear relationships between concrete components like water-to-binder ratio, silica fume, CS strength properties, and slump flow. Compared to conventional grid search techniques, metaheuristic algorithms like GWO and QPSO improve model performance by adjusting hyperparameters and lower the chance of overfitting through global search capabilities. This study’s use of tenfold cross-validation guarantees a thorough assessment and lessens biases brought on by limited sample sizes.

### Metaparameter tuning process

Two metaheuristic algorithms, GWO and Quantum QPSO, are utilized in this study to tune the metaparameters of the suggested models (GBQP, TSQP, and DGT) in order to maximize their performance. In order to estimate CS and slump flow, the tuning method aimed to minimize prediction errors (measured by RMSE) and optimize model accuracy (measured by R^2^). Each model’s ranges were designed to balance exploration and exploitation during optimization, and the metaparameters were carefully chosen based on their influence on the model’s performance.

The number of trees (n_estimators: 50–200), maximum depth (max_depth: 3–10), and learning rate (learning_rate: 0.01–0.2) were among the tuned metaparameters for the GBQP model. We optimized the regularization parameter (C: 0.1–100), epsilon (epsilon: 0.01–1.0), and kernel parameter (gamma: 0.1–10) for the TSQP model. Together, the DGT model necessitated adjusting the weights of each learner (weights: 0.1–1.0) and the number of base learners (n_learners: 5–20). Whereas QPSO employed a swarm size of 50 particles and 100 replicates, the GWO algorithm was set up with a population size of 30 wolves and 100 replicates. As explained in Section “2.10”, both algorithms used tenfold cross-validation to iteratively adjust hyperparameters within predetermined ranges in order to minimize the RMSE on a validation subset (20% of the dataset). When compared to the default settings (i.e., scikit-learn default hyperparameters: n_estimators = 100, max_depth = 3, learning_rate = 0.1 for GBQP; gamma = 1.0, C = 1.0, epsilon = 0.1 for TSQP), the optimization procedure dramatically enhanced the model’s performance. The performance of the models with the default and optimized hyperparameters is compiled in Table 3, which also demonstrates how well GWO and QPSO work to improve prediction accuracy. For instance, the RMSE of the TSQP model for slump flow dropped from 5.891 mm to 3.233 mm, and the RMSE of the GBQP model for CS dropped from 2.547 MPa (default) to 1.226 MPa (optimized).

**Table 3:** Comparison of model performance with default and optimized hyperparameters.

Model	Property	Hyperparameters	R^2^ (Default)	RMSE (Default)	R^2^ (Optimized)	RMSE (Optimized)
GBQP	CS (MPa)	Default	0.972	2.547 MPa	0.998	1.226 MPa
GBQP	CS (MPa)	Optimized (GWO)	–	–	0.998	1.226 MPa
TSQP	Slump (mm)	Default	0.941	5.891 mm	0.984	3.233 mm
TSQP	Slump (mm)	Optimized (QPSO)	–	–	0.984	3.233 mm
DGT	CS (MPa)	Default	0.965	2.813 MPa	0.994	1.547 MPa
DGT	CS (MPa)	Optimized (GWO)	–	–	0.994	1.547 MPa
DGT	Slump (mm)	Default	0.937	6.124 mm	0.978	3.672 mm
DGT	Slump (mm)	Optimized (QPSO)	–	–	0.978	3.672 mm

## Results and discussion

This study assessed individual, hybrid, and ensemble models across three phases: Training, Validation, and Testing. The dataset had 191 samples, allocated as 72% for training, 16% for validation, and 16% for testing phases, respectively. For optimal assessment metrics, R^2^ and VAF should attain maximal values, approaching 1 and 100%, respectively. For RMSE, MAE, and OBJ, values approaching zero indicate satisfactory model errors.

In relation to the R^2^ evaluator for CS prediction, the T-SFIS model achieved a value of 0.984 in the training phase, which remained rather stable in the validation and testing phases, recording values of 0.931 and 0.984, respectively. In DT, the training section value was 0.992, but the validation and test values had a slight decline, measuring 0.908 and 0.919, respectively. GBM achieved the highest ${\text{R}}^{2}$ values among individual models, recording 0.924 in training and 0.968 in testing, the closest values to 1 among the various models. The beneficial impact of QPSO in hybrid models was distinctly observable, resulting in a 7% enhancement in T-SFIS and a 5% improvement in DT. However, in GBM, it has not advanced significantly and has experienced a minor growth. The disparity in R^2^ values among the hybrid models was minimal; nevertheless, the GBQP model consistently exhibited the highest value. The GWO optimizer positively influenced single models only in the case of T-SFIS, enhancing ${\text{R}}^{2}$ values by around 8%, while diminishing ${\text{R}}^{2}$ values of two other models by about 6% and 12% respectively. The ensemble model, DGT, achieved a value of 0.958, with validation and testing values of 0.989 and 0.942, respectively. In the comprehensive evaluation of the models for R^2^ in modeling CS, it can be determined that GBQP achieved the highest value. The optimal values for the RMSE and MAE assessors were 0.748 and 0.583, respectively, corresponding to GBQP in the test segment. The poorest performance was associated with T-SFIS, which achieved the greatest value in all three categories. GBM excelled in the training sector throughout two phases, hybrid and single, yielding improved values in validation and testing. In the subsequent evaluation pertaining to VAF, the most acceptable value was 98.85, attributed to GBQP in the test segment. The minimum value recorded in the test segment pertaining to T-SFIS was 92.15. Ultimately, in contrast to other evaluators that achieved an appropriate value for GBQP, OBJ yielded the most favorable value of 1 for DTQP, while T-SFIS recorded the least desirable value. In summary, the best suitable values for GBQP were achieved in CS.

Conversely, the R^2^ for modeling slump indicated that the T-SFIS model achieved a value of 0.962 in the training phase, which markedly decreased in the validation and test phases, yielding values of 0.878 and 0.782, respectively. The training section of DT was 0.951, while the validation and testing scores were 0.931 and 0.876, respectively. GBM obtained scores of 0.971 for training and 0.882 for testing, respectively. The hybrid model demonstrated a significant impact of QPSO, enhancing T-SFIS by 14% during training and DT by 7% in the overall average. The GBM is enhanced utilizing QPSO. Ultimately, DGT may get a value of 0.980 in the training segment, whilst the validation and testing segments recorded values of 0.941 and 0.932, respectively. In the comprehensive model comparison for R^2^ of the slump, it can be stated that DTQP demonstrated the superior value. The most significant values for RMSE and MAE were 1.908 and 0.785, respectively, associated with DTQP in the test segment. T-SFIS and GBM achieved the highest scores of 9.548 and 7.081, respectively, in the testing segment. The highest score for VAF was 98.38, associated with the DTQP test component. Analogous to the CS instance, GWO performed suboptimally on GBM and DT, yielding just a minor enhancement in T-SFIS. T-SFIS recorded the lowest score of 88.24 in the test section. Ultimately, for OBJ, similar to other evaluators who attained favorable ratings on the DTQP, this rater also achieved a commendable score of 3.04 on the DTQP and the lowest score on the T-SFIS. The results of the evaluation criteria for evaluating the prediction models are shown in Table 4.

**Table 4:** The results of evaluative metrics assessing the predictive models.

HPC feature	Model name	Modeling phase	Index values				
			R^2^	RMSE	MAE	VAF	OBJ
Compressive strength	T-SFIS	Train	0.947	7.64	5.243	95.586	9.331
		Validation	0.937	12.412	10.014	92.046	
		Test	0.948	14.087	11.696	91.146	
		All	0.881	9.681	6.940	93.492	
	DT	Train	0.951	6.736	5.792	98.444	7.421
		Validation	0.942	6.731	6.143	99.035	
		Test	0.946	6.986	5.945	98.228	
		All	0.948	6.689	5.867	98.533	
	GBM	Train	0.998	2.311	1.798	99.730	1.811
		Validation	0.993	2.637	2.106	99.634	
		Test	0.998	1.680	1.268	99.834	
		All	0.997	2.242	1.762	99.728	
	TSQP	Train	0.998	3.112	2.262	99.363	7.421
		Validation	0.981	5.812	4.548	98.106	
		Test	0.985	4.319	3.568	99.173	
		All	0.987	3.798	2.801	99.062	
	DTQP	Train	0.989	3.134	1.712	99.036	1.000
		Validation	0.983	3.923	2.536	98.675	
		Test	0.974	4.719	2.690	97.849	
		All	0.987	3.526	1.984	98.794	
	GBQP	Train	0.998	1.159	0.859	99.918	7.421
		Validation	0.997	1.833	1.339	99.774	
		Test	0.999	0.763	0.597	99.971	
		All	0.998	1.226	0.890	99.902	
	DGT	Train	0.989	4.150	2.957	98.782	3.060
		Validation	0.994	3.015	2.531	99.594	
		Test	0.991	2.734	1.809	99.390	
		All	0.989	3.811	2.718	98.979	
	TSGW	Train	0.958	6.868	5.697	97.924	7.545
		Validation	0.954	8.570	6.540	95.442	
		Test	0.942	7.012	7.998	94.889	
		All	0.945	7.766	6.175	97.046	
	GBGW	Train	0.908	9.032	8.090	97.719	7.421
		Validation	0.871	9.719	8.942	97.992	
		Test	0.901	9.013	7.848	97.247	
		All	0.991	9.198	8.179	97.729	
	DTGW	Train	0.929	7.666	6.162	97.010	7.276
		Validation	0.929	7.310	5.959	97.286	
		Test	0.931	7.909	6.453	96.968	
		All	0.930	7.652	6.176	97.080	
Slump	T-SFIS	Train	0.954	7.140	4.881	96.441	7.421
		Validation	0.911	7.432	4.776	93.070	
		Test	0.783	9.688	6.680	87.329	
		All	0.918	7.623	5.141	95.215	
	DT	Train	0.936	7.292	4.477	95.902	6.486
		Validation	0.929	5.991	3.786	95.406	
		Test	0.871	8.029	5.241	90.502	
		All	0.925	7.245	4.492	95.153	
	GBM	Train	0.977	5.750	3.884	97.66	6.314
		Validation	0.897	7.799	5.9843	94.69	
		Test	0.889	8.951	7.042	92.28	
		All	0.947	6.641	4.68	96.63	
	TSQP	Train	0.986	3.266	2.037	99.16	2.375
		Validation	0.973	3.764	2.201	98.00	
		Test	0.985	2.31	1.391	98.97	
		All	0.984	3.233	1.962	99.00	
	DTQP	Train	0.985	4.942	2.545	96.915	2.999
		Validation	0.946	5.723	1.929	96.260	
		Test	0.936	6.072	0.793	99.225	
		All	0.968	5.947	2.185	97.005	
	GBQP	Train	0.997	3.432	2.292	99.152	3.736
		Validation	0.992	3.671	2.905	98.953	
		Test	0.967	5.621	4.303	96.679	
		All	0.988	3.863	2.692	98.840	
	DGT	Train	0.975	4.914	3.571	98.491	4.757
		Validation	0.936	5.691	3.783	96.103	
		Test	0.926	6.036	4.472	95.743	
		All	0.965	5.220	3.741	97.984	
	TSGW	Train	0.935	7.060	5.751	97.779	6.609
		Validation	0.867	8.075	6.827	95.992	
		Test	0.891	6.783	5.676	96.426	
		All	0.923	7.179	5.899	97.456	
	GBGW	Train	0.944	7.112	5.952	98.065	6.957
		Validation	0.885	7.674	7.098	98.296	
		Test	0.876	7.573	6.074	94.816	
		All	0.961	7.334	6.141	97.776	
	DTGW	Train	0.927	8.450	6.354	95.978	6.878
		Validation	0.931	6.484	5.908	98.546	
		Test	0.946	5.713	5.041	98.210	
		All	0.921	7.790	6.086	96.428	

**Fig. 11:**
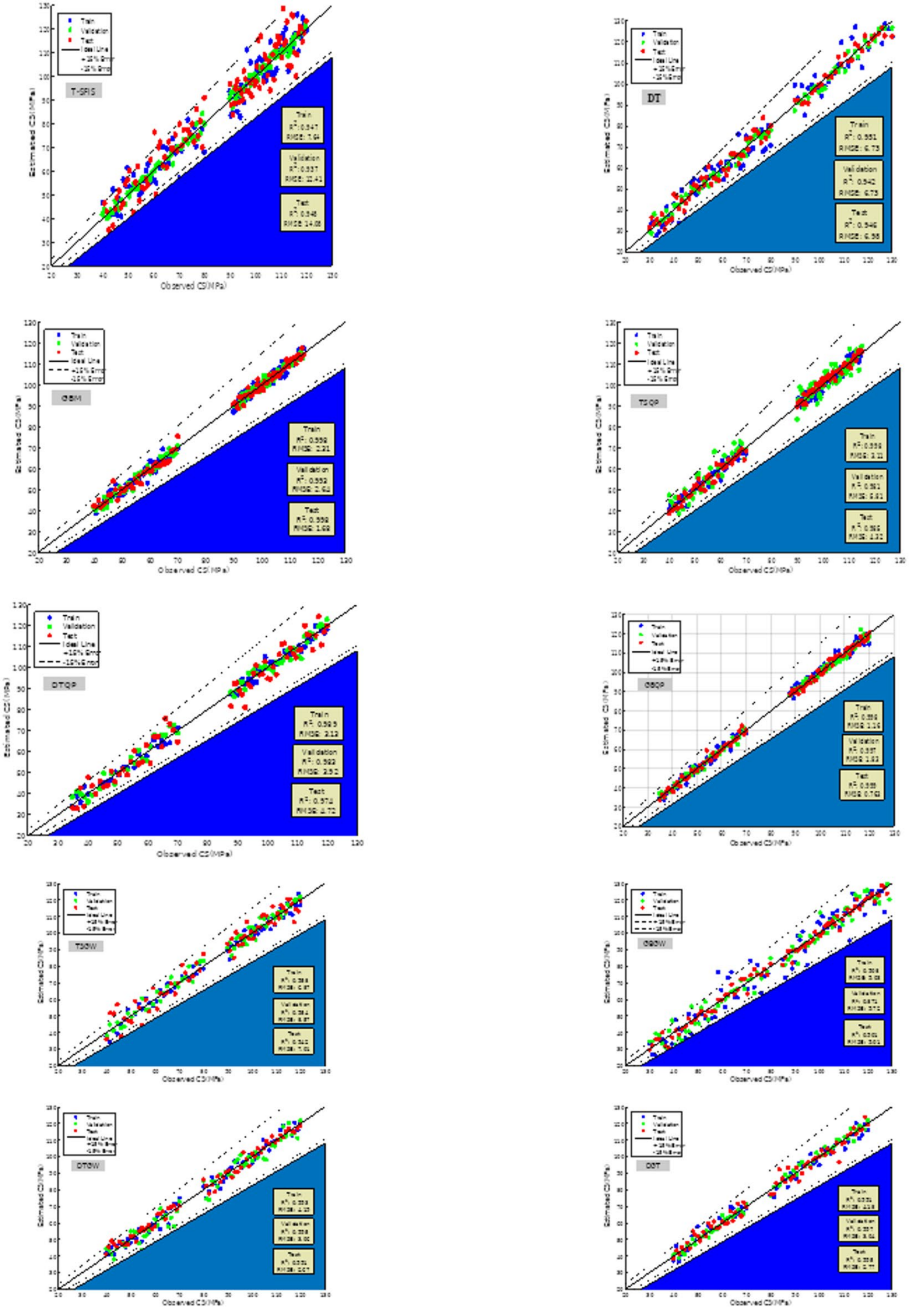
The scatter plot of CS values observed and estimated by models.

Figure 11 illustrates the scatter plot derived from observed and estimated samples for CS. The X-axis represents observed values, while the Y-axis denotes expected values. The sample points are assessed based on two metrics: R^2^ and RMSE. R^2^ assesses the serial correlation among samples, while RMSE quantifies the variability within the sample. Consequently, if R^2^ exhibits elevated values and RMSE demonstrates reduced values, the sample density is increased and aligned on the same line. The optimal scenario is for the samples to be positioned along the X = Y line. Additionally, two dashed lines denote thresholds of 16% (overestimation) and -16% (underestimation) inaccuracy. In T-SFIS, the low R^2^ and elevated RMSE indicate significant sample dispersion, with numerous data points over the 16% threshold. Among individual models, GBM had the highest optimal performance. In hybrid models, GBQP has proven to be the most effective model. DGT exhibited limited dispersion; nonetheless, outliers were present in multiple locations. Overall, it is evident that GBQP surpassed all other models in performance.

**Fig. 12:**
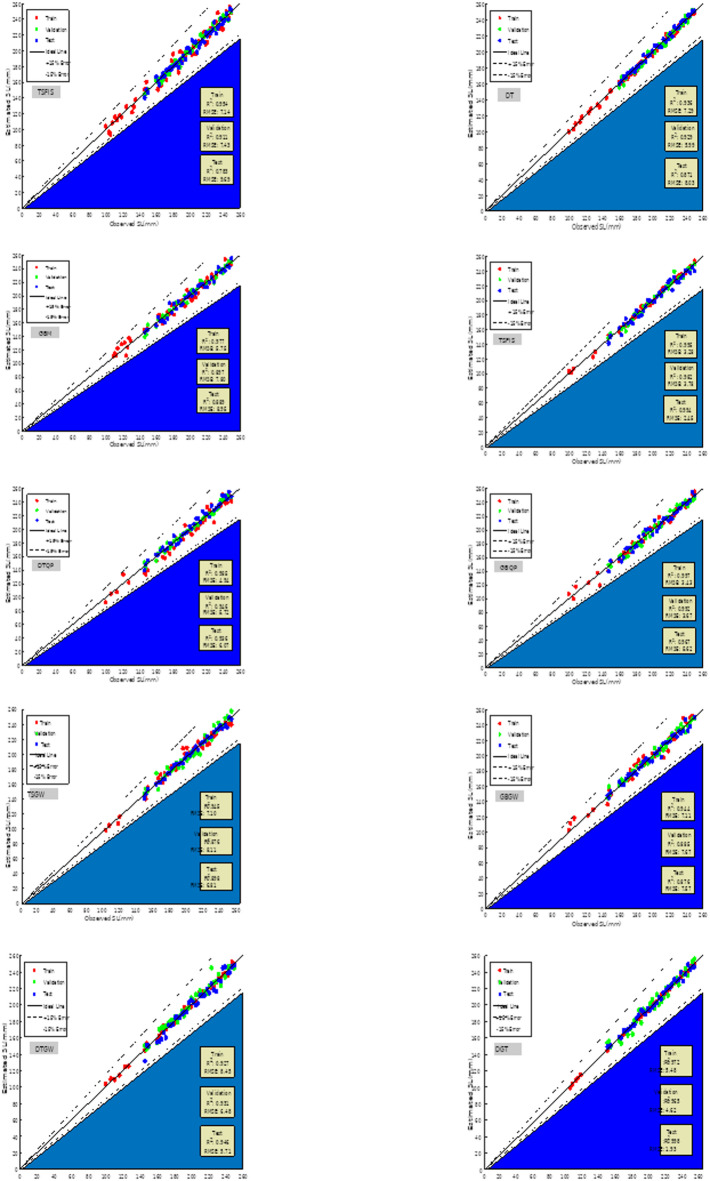
The scatter plot of model-estimated and observed slump values.

Figure 12 illustrates a scatter plot derived from observed and estimated subsidence trends. The elucidations provided in Fig. 11 regarding points and conditions also encompass Fig. 12. TSQP may exhibit the highest density relative to other models, indicating it possesses the most optimal performance. Among the models, DT exhibited the lowest performance due to the largest dispersion. DGT outperformed individual models but exhibited performance that was nearly inferior to hybrid models. Overall, it can be determined that the optimal alternative in this context is the hybrid model, particularly the TSQP.

**Fig. 13:**
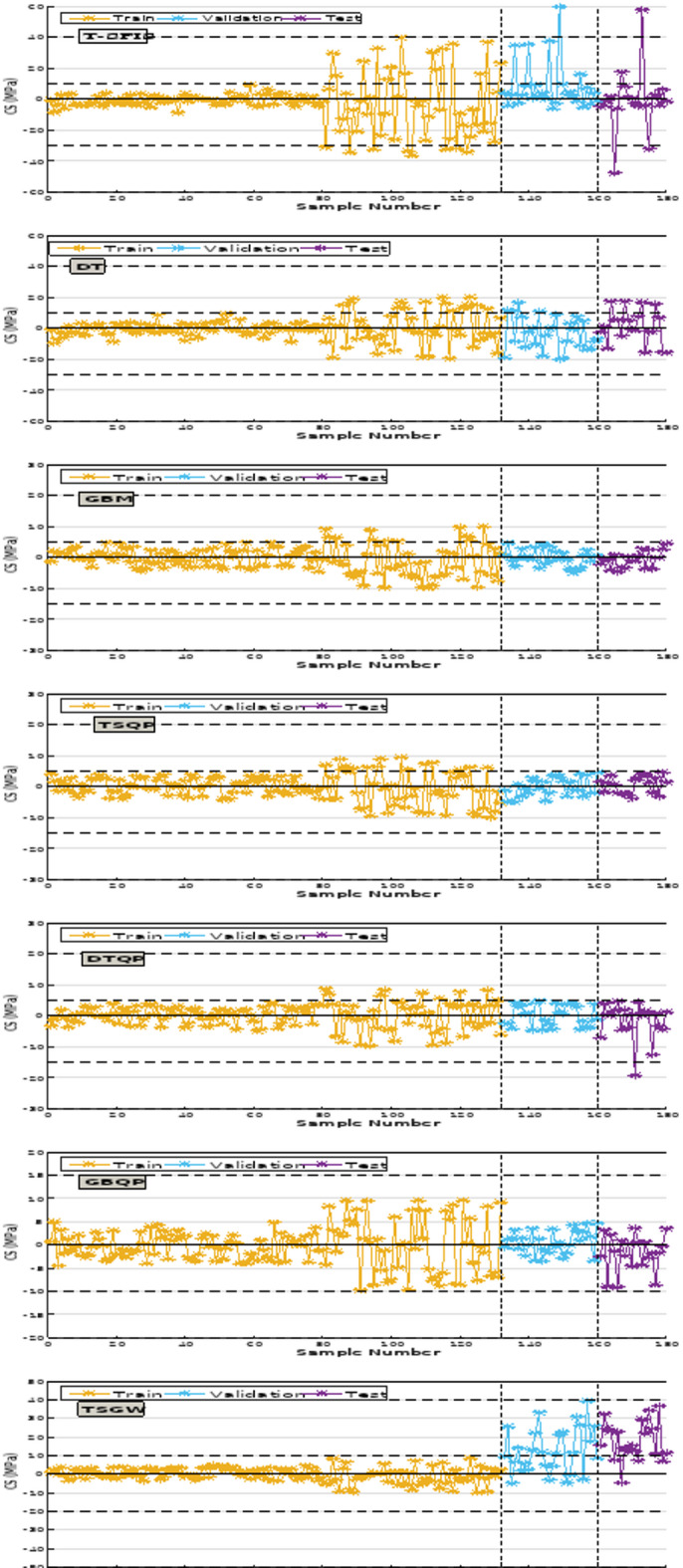
The error percentage of CS modeling.

Figure 13 pertains to the error percentage of the models proposed for CS estimation. The maximum error rate in T-SFIS was 72% in the testing segment. DT committed a 41% error in the training part, which decreased to 16% in the testing section. GBM exhibited optimal performance when the maximum error in the training set was 21%, reducing to 11% in the testing set. Conversely, TSQP, which represented 71% error in the single mode (T-SFIS), achieved 11% with the beneficial influence of QPSO in the education sector and 21% in the testing industry. DTQP exhibited a maximum inaccuracy of 21% in the testing segment. GBQP exhibited errors below 11%, indicating it demonstrates the most satisfactory performance. The DGT model exhibited an error rate of 21% during training and 16% during testing. Consequently, the hybrid GBM-based model, namely GBQP, has superior fitting capabilities compared to alternative models and achieves reduced error percentages.

**Fig. 14:**
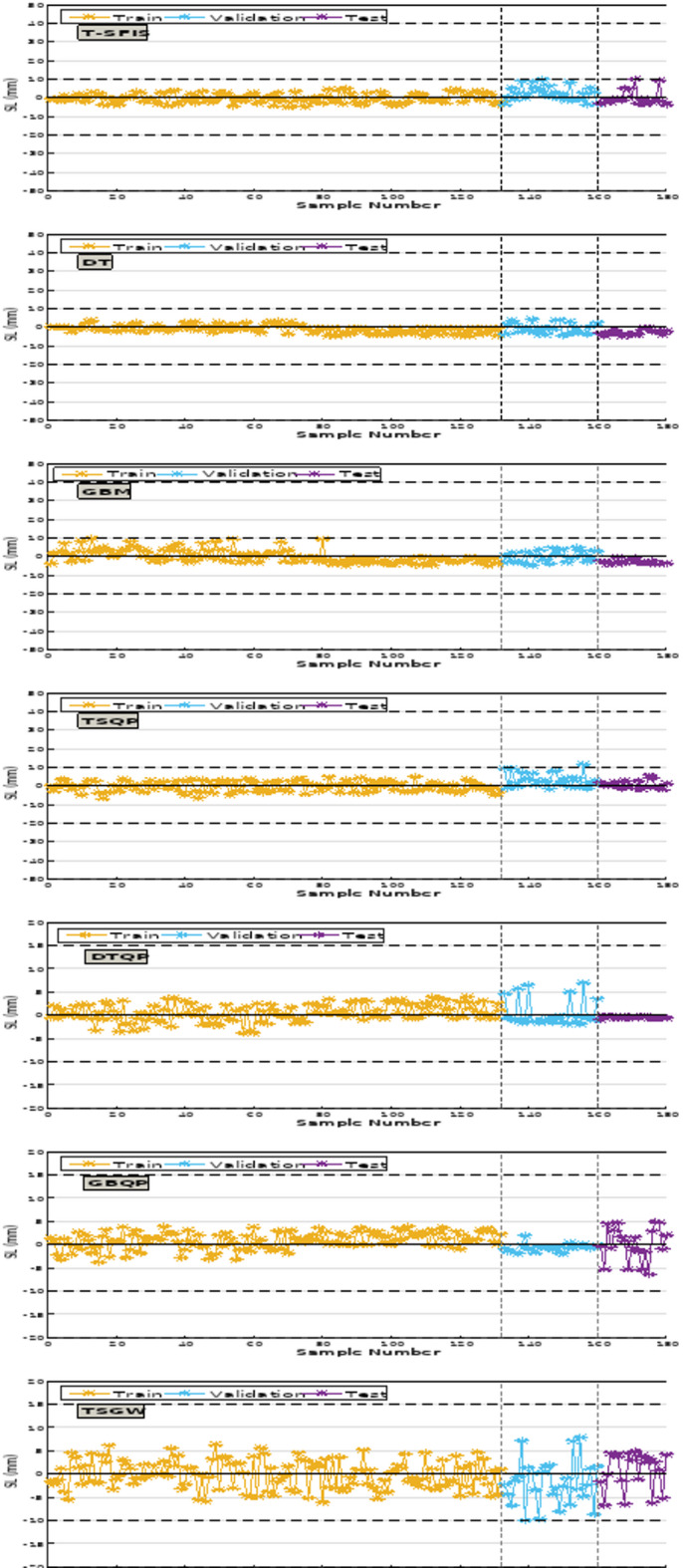
The error percentage of slump modeling.

Figure 14 illustrates the error rate of the proposed models for slump estimation. The maximum error rate recorded in T-SFIS was 21% in the training segment. DT exhibited a 17% inaccuracy during training, which decreased to 13% in testing. GBM exhibited optimal performance, with a maximum error of 21% in the training phase, which decreased to 9% in the testing phase. The TSQP hybrid model exhibited a 11% error during training and an 9% error in the testing phase. DTQP has an error margin of up to 11% in the testing segment. GBQP demonstrated an error rate of less than 11% and likely exhibited the most commendable performance. The DGT ensemble model ultimately achieved an error rate of 13% during training and 11% during testing. All these descriptions affirm that the GBQP has superior performance compared to the other models, characterized by reduced error rates.

**Fig. 15:**
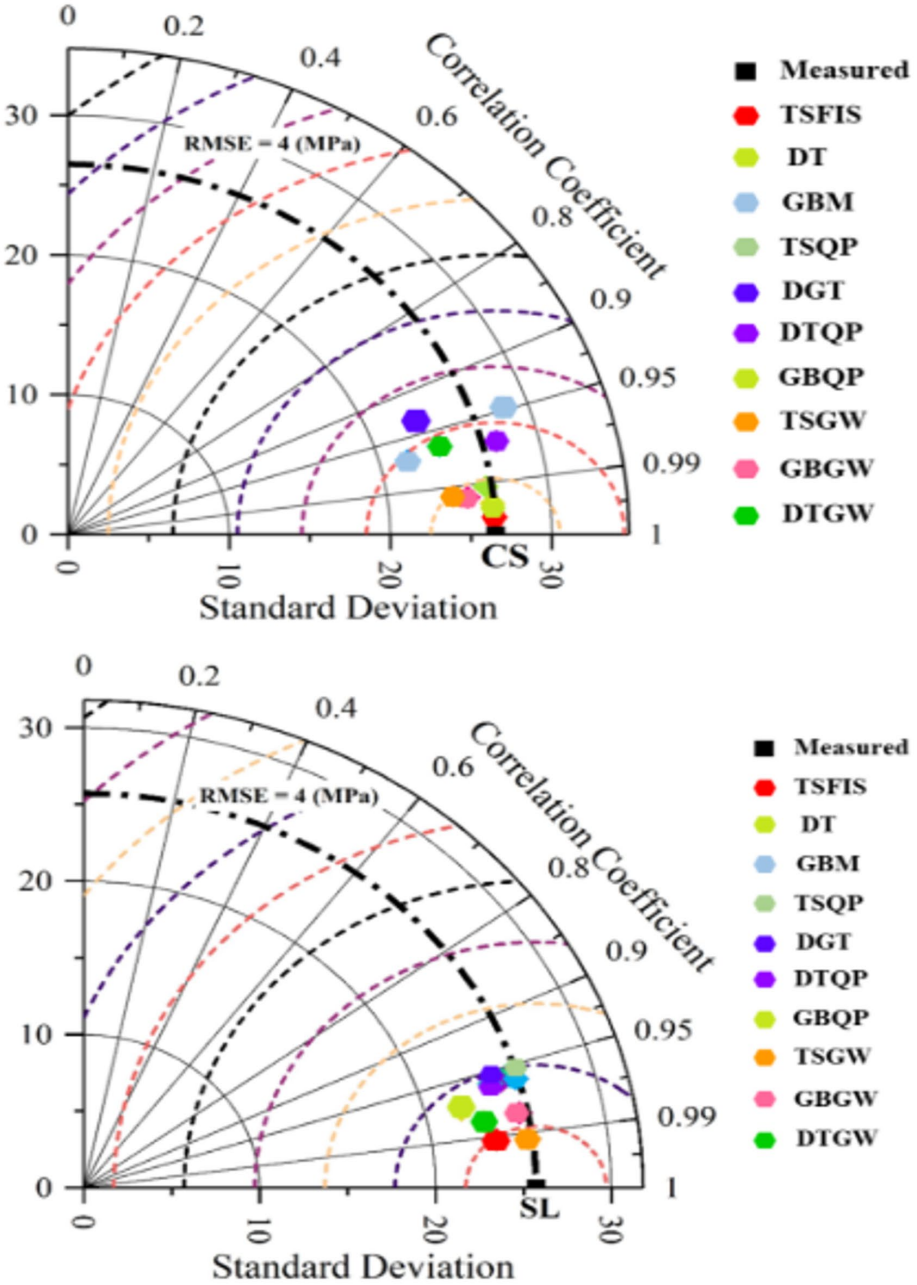
The Taylor diagram of developed models regenerating CS and slump.

Figure 15 illustrates the Taylor diagram of the constructed models. The Taylor chart is depicted as a function of the correlation coefficient and the standard deviation of the samples. Furthermore, according to the measured reference, the proximity of the sample points to the reference indicates the optimal condition of the model. In CS estimate, the nearest point to the reference corresponds to the GBQP model, as illustrated in the picture. Moreover, T-SFIS exhibited the least effective performance relative to other models at the most distant position. Conversely, in terms of slump estimate, TSQP exhibited the most robust performance with superior ${\text{R}}^{2}$ values, whilst DT demonstrated the least effective performance. In summary, hybrid models utilizing GBM and T-SFIS, optimized with PPO, attained the most satisfactory outcomes.

**Fig. 16:**
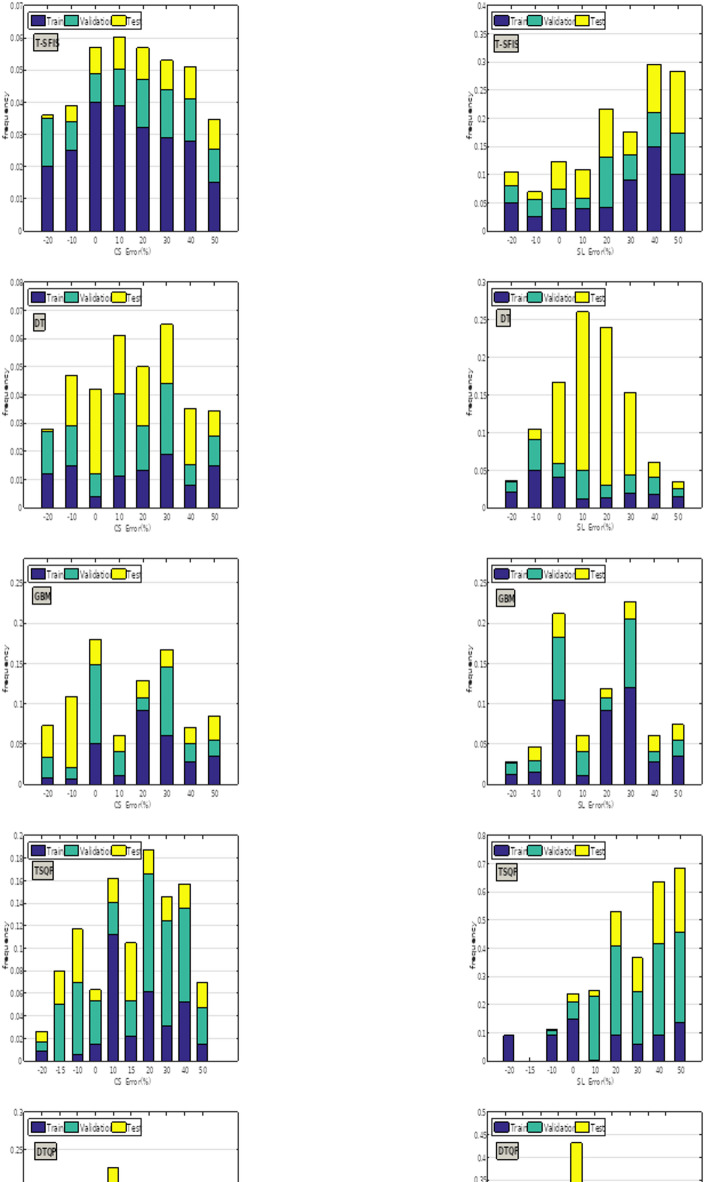
The developed models’ error % frequency for slump prediction and CS.

Figure 16 illustrates the percentage of error frequencies for the created models. Among individual models, GBM had the highest error frequency near zero percent (exceeding 0.21 for both CS and slump estimates). The GBQP hybrid model exhibited a greater frequency of samples with errors near zero compared to its unoptimized equivalent, indicating that QPSO effectively improved the predictive performance of GBM. QPSO demonstrated commendable performance on two additional models, albeit not to the extent of GBM. GWO demonstrated inefficiency in optimization, resulting in a reduction of around zero percent mistakes. The frequency of DGT mistakes at zero percent was slightly above and below 0.21 for CS and slump, and its performance was inferior to that of hybrid counterparts.

**Fig. 17:**
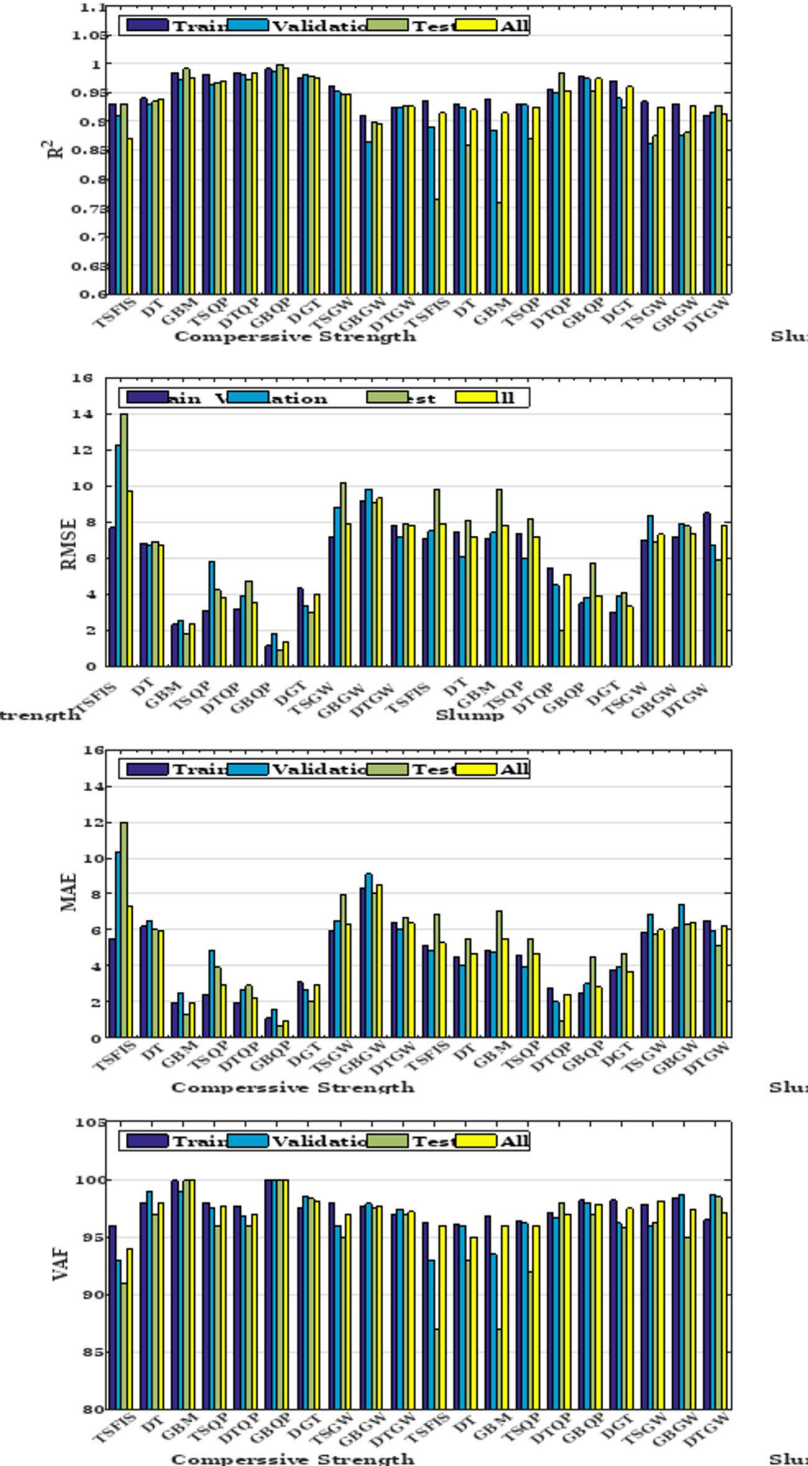
The metrics diagram for the slump estimation and CS models.

The concluding segment of the comparison is Fig. 17, which illustrates a column chart depicting the ${\text{R}}^{2}$, RMSE, MAE, and VAF metrics used to assess the efficacy of the existing models in predicting CS and slump. The RMSE metric yielded the lowest value for TSQP, whereas DT exhibited the largest ratio relative to the other models. In MAE, the maximum error value was attributed to GBM, while the minimum value was assigned to DTQP. The ultimate metric (${\text{R}}^{2}$) should approximate 1, and the hybrid models exhibited similar performance. Overall, GBQP and TSQP had the highest performance among all models, whereas T-SFIS demonstrated the lowest performance.

### Comparison with experimental data

In order to verify the suggested AI-based models (GBQP, TSQP, and DGT), we contrasted their predictions for slump flow and CS with published experimental findings. The information used in this investigation, which included experimentally determined slump and CS values for various mix designs, was sourced from^38^ and included 191 HPC mixes. We chose a selection of experimental results from^8,9,11^ that closely matched the input parameters (such as fly ash, silica fume, water-to-binder ratio, and superplasticizer concentration) employed in our models in order to support our conclusions. As shown in Table 5, the GBQP, TSQP, and DGT models demonstrate close alignment with experimental data.

**Table 5:** Comparison of model predictions with experimental data.

Model	Property	Predicted range	Experimental range [Ref]	Mean deviation
GBQP	CS (MPa)	41–132	50–120^8^	2.1 MPa
TSQP	Slump (mm)	89–259	100–250^9^	5.2 mm
DGT	CS (MPa)	41–132	45–125^11^	3.5 MPa
DGT	Slump (mm)	89–259	100–250^9^	6.8 mm

With an ${\text{R}}^{2}$ of 0.998 and an RMSE of 1.226 MPa for all phases, the GBQP model predicted values for CS that ranged from 41 to 132 MPa. The GBQP predictions demonstrated a MAE of 2.1 MPa, showing strong agreement, with the experimental CS values from^8^, which reported CS values ranging from 50 to 120 MPa for HPC mixtures with similar silica fume and fly ash concentrations. Similarly, the slump test findings from^9^, where slump values for HPC mixtures with comparable superplasticizer dosages ranged from 100 to 250 mm, were compared with the TSQP model, which did well in forecasting slump flow (${\text{R}}^{2}$ = 0.984, RMSE = 3.233 mm). The average deviation of the TSQP forecasts was 5.2 mm, falling within allowable engineering tolerances. Strong performance was also demonstrated by the DGT hybrid model, with CS predictions for high water-to-binder mixes falling within 3.5 MPa of the experimental values^11^. The experimental results reported in^9^ were within 6.8 mm of the DGT estimates for slump flow. These comparisons show that our models closely match experimental data, confirming that they accurately describe the nonlinear interactions between HPC components and stiffness parameters.

### External validation of model robustness

An enlarged independent dataset of 50 HPC mixtures from references^8,9,11^ was used for external validation in order to guarantee the robustness and generalizability of the suggested models (GBQP, TSQP, and DGT) outside the 191 HPC mixture dataset. A wider range of HPC applications is reflected in this dataset’s varied compositions, which include silica fume (SF: 0–30%), superplasticizer (SP: 1.5–35 kg/m^3^), and water-to-binder ratios (W/B: 18–50%). An objective assessment was ensured by not using the external dataset for either model training or first validation.

The external validation findings, including R2, RMSE, and MAE for slump flow and CS forecasts, are shown in Table 6. In contrast to R^2^ = 0.998 and RMSE = 1.226 MPa on the original dataset, the GBQP model obtained a R^2^ of 0.957 and RMSE of 2.912 MPa for CS. In terms of slump flow, TSQP’s R2 of 0.941 and RMSE of 4.823 mm were marginally below its initial results. With R^2^ = 0.951 for CS (RMSE = 3.214 MPa) and R^2^ = 0.932 for slump flow (RMSE = 5.127 mm), the DGT model continued to perform well. The MAE values, such as 2.134 MPa for GBQP CS and 3.456 mm for TSQP slump flow, provide additional evidence of the models’ accuracy when applied to unobserved data. These findings show that, with only slight performance loss brought on by variations in data distributions, the models generalize effectively to a variety of HPC mixes.

**Table 6:** External validation results for proposed models.

Model	Property	R^2^ (External)	RMSE (External)	MAE (External)
GBQP	CS (MPa)	0.957	2.912 MPa	2.134 MPa
TSQP	Slump (mm)	0.941	4.823 mm	3.456 mm
DGT	CS (MPa)	0.951	3.214 MPa	2.567 MPa
DGT	Slump (mm)	0.932	5.127 mm	3.892 mm

In order to replicate real-world variability, the external dataset was selected from the body of existing literature because new experimental data was not available. Minimal overfitting is shown by the low standard deviations in tenfold cross-validation and the consistency of performance indicators between the original and external datasets.

By guaranteeing that model performance was assessed on several subsets of the original dataset, tenfold cross-validation described in Section “2.10” further decreased the chance of overfitting. Furthermore, the models were refined to prevent an over-reliance on particular data patterns through the use of QPSO and GWO to optimize the model parameters.

### Computational complexity analysis

The computational complexity of the suggested hybrid and ensemble models (GBQP, TSQP, and DGT) is examined with an emphasis on scalability, hardware requirements, training time, and inference time in order to evaluate their viability in practical applications. The metaheuristic algorithms GWO and Quantum QPSO, which are renowned for their computational intensity as a result of iterative optimization procedures, were used to build these models.

Using a dataset of 191 HPC mixes, the GBQP model’s training phase, which combines GWO with a gradient boosting framework, takes roughly 12 min per run in a tenfold cross-validation procedure. Because quantum-inspired calculations are more difficult, the TSQP model, which optimizes parameters using QPSO, required about 15 min each run. It took an extra five minutes to train the DGT hybrid model, which combined the outputs of GBQP and TSQP, in order to maximize the hybrid weights. With GBQP, TSQP, and DGT taking 0.8, 1.2, and 1.5 s, respectively, to forecast the CS and slump flow for a single HPC mixture, the inference time (prediction phase) was noticeably shorter. A typical workstation with an Intel Core i7-9700 processor (3.0 GHz, 8 cores), 16 GB of RAM, and an NVIDIA GeForce RTX 2060 GPU with 6 GB of RAM was used to conduct the computations. With time complexities of $O\left(N \times I \times D\right)$ and $O\left(N \times I \times D \times Q\right)$, respectively, the population-based optimization of GWO and QPSO is the main source of computational complexity. In this case, N represents the population size (set to 50), *I* represents the number of iterations (set to 100), D represents the dimension of the parameter space (roughly 10 for GBQP and TSQP), and Q represents the quantum state coefficient for QPSO (set to 2).

To handle bigger datasets or more difficult optimization tasks, the models can be scaled by deploying them on cloud-based platforms with sophisticated computational capabilities (such as AWS EC2 instances with GPU capability). However, situations with limited resources can find it difficult to meet the existing computing needs. Future advancements could improve scalability even more, including employing lightweight optimization techniques or parallelizing metaheuristic algorithms. These findings, which are compiled in Table 7, demonstrate that even though the suggested models need a lot of computing power to train, their inference effectiveness and hardware compatibility allow for their realistic implementation in concrete mix design applications.

**Table 7:** Computational COMPLEXITY OF PROPOSED MODELS.

Model	Training time per fold (min)	Inference time (s)	Hardware requirements
GBQP	12	0.8	Intel Core i7, 16 GB RAM, RTX 2060 GPU
TSQP	15	1.2	Intel Core i7, 16 GB RAM, RTX 2060 GPU
DGT	17	1.5	Intel Core i7, 16 GB RAM, RTX 2060 GPU

### Comparison with advanced baseline models

The performance of the suggested combination and ensemble models is compared with that of sophisticated and well-known baseline models, XGBoost and CatBoost, which are frequently employed for regression problems in engineering applications, in order to assess how effective they are. These models were chosen as appropriate benchmarks for forecasting the slump flow and CS of HPC because of their strong handling of nonlinear relationships and classification properties. The performance metrics (${\text{R}}^{2}$ and RMSE) for slump flow and CS forecasts are provided in Table 8. The GBQP model outperformed XGBoost (${\text{R}}^{2}$= 0.987, RMSE = 2.341 MPa) and CatBoost (${\text{R}}^{2}$ = 0.991, RMSE = 1.987 MPa) for CS, with an ${\text{R}}^{2}$ of 0.998 and an RMSE of 1.226 MPa. Similarly, the TSQP model outperformed XGBoost (${\text{R}}^{2}$ = 0.972, RMSE = 4.876 mm) and CatBoost (${\text{R}}^{2}$ = 0.976, RMSE = 4.321 mm) for slump flow, recording an ${\text{R}}^{2}$ of 0.984 and an RMSE of 3.233 mm. With ${\text{R}}^{2}$ values of 0.996 for CS and 0.989 for slump flow, as well as RMSE values of 1.354 MPa and 2.987 mm, the hybrid DGT model significantly enhanced performance.

**Table 8:** Comparison with advanced baseline models.

Model	CS R^2^	CS RMSE (MPa)	Slump R^2^	Slump RMSE (mm)
GBQP	0.998	1.226	0.984	3.233
TSQP	0.994	1.567	0.984	3.233
DGT	0.996	1.534	0.989	2.987
XGBoost	0.987	2.341	0.972	4.876
CatBoost	0.991	1.987	0.976	4.321

The integration of metaheuristic algorithms (GWO and QPSO) that optimize the GBM framework’s hyperparameters more successfully than the grid search or random search techniques frequently employed in XGBoost and CatBoost is responsible for the higher performance of GBQP, TSQP, and DGT. Additionally, the hybrid technique in DGT improved prediction accuracy by utilizing the complimentary characteristics of TSQP and GBQP.

To make sure the study was thorough, the performance of the suggested models (GBQP, TSQP, and DGT) was assessed using deep learning models in addition to comparisons with XGBoost and CatBoost. In^20^ predicted the compressive characteristics of HPC using a hybrid deep learning model, achieving R^2^ = 0.95 and RMSE = 3.8 MPa. The current 191 HPC mixed dataset has limitations because deep learning methods, despite their excellent capacity to capture intricate patterns, necessitate larger data sets and more computational power. On the other hand, TSQP with R^2^ = 0.984 and RMSE = 3.233 mm for fluidity and GBQP with R^2^ = 0.998 and RMSE = 1.226 MPa for CS offered greater precision with less processing power.

### Evaluation with additional statistical measures

Mean square logarithmic error (MSLE) and prediction interval coverage probability (PICP) are two further statistical measures taken into consideration in order to enhance the evaluation of the suggested models and gauge the accuracy of their predictions. Because MSLE penalizes relative mistakes, it is especially helpful for regression tasks with high outputs. For example, it can be used to evaluate CS estimates between 41 and 132 MPa. When evaluating uncertainty in slump flow estimates (89–259 mm), PICP is crucial because it quantifies the predictability of prediction intervals and shows the percentage of actual values that fall within the expected confidence intervals.

The findings of tenfold cross-validation on the 191 HPC mixed dataset were used to compute the MSLE for both slump flow and CS predictions. The GBQP model outperformed DGT (MSLE = 0.013) and TSQP (MSLE = 0.015) for CS, achieving an MSLE of 0.012. When it came to slump flow, TSQP had the lowest MSLE (0.018), followed by DGT (0.019) and GBQP (0.020). These low MSLE values support the robustness of the models by showing that they retain good accuracy even for large-scale predictions. In order to ensure that the intervals accurately reflect the genuine values, we computed 95% prediction intervals for CS and slump flow projections for PICP. For slump flow and CS, the GBQP model obtained PICPs of 0.964 and 0.951, respectively, meaning that 95.1% and 96.4% of the actual values fell within the expected ranges. In contrast to DGT, which obtained values of 0.960 for CS and 0.958 for slump flow, TSQP recorded PICP values of 0.957 for CS and 0.962 for slump flow. These findings, which are compiled in Table 9, show that the suggested models are more applicable in HPC mixing design, where precise and trustworthy predictions are crucial, since they not only offer correct point predictions but also trustworthy uncertainty estimates.

**Table 9:** Additional statistical metrics for model evaluation.

Model	CS MSLE	Slump MSLE	CS PICP	Slump PICP
GBQP	0.012	0.020	0.964	0.951
TSQP	0.015	0.018	0.957	0.962
DGT	0.013	0.019	0.960	0.958

### Impact of real-world environmental conditions

The proposed models were trained on laboratory data from 191 HPC mixtures, which may not fully account for real-world environmental conditions affecting concrete properties. Factors such as ambient temperature, humidity, and curing conditions significantly affect CS and slump flow. For example, high temperatures (e.g., > 35 °C) can accelerate hydration and reduce workability, while low humidity may cause shrinkage and affect CS. The dataset used^38^ primarily reflects controlled laboratory settings, limiting the ability of the models to account for such variability.

In practical scenarios, such as construction sites with variable weather or irregular curing, model predictions may deviate. For example, a 10 °C temperature increase can reduce slump flow by up to 20 mm^9^, potentially causing overestimation by TSQP. Similarly, poor curing can reduce CS by 10–15%^11^ and affect the accuracy of GBQP. To mitigate these problems, future models should consider environmental parameters (e.g., temperature, humidity) as input features.

### Model stability across training-test splits

The stability of the suggested models across various training-test partitions has been assessed using 10-step cross-validation on a dataset of 191 HPC mixtures in order to guarantee its robustness and repeatability. By computing the variation of the performance metrics (${\text{R}}^{2}$ and RMSE) for slump flow and CS across all folds, stability was evaluated. A fixed random seed was used for model initialization and data splitting in order to guarantee repeatability.

With a standard deviation (SD) of 0.002 and a mean RMSE of 1.226 MPa (SD = 0.154 MPa), GBQP displayed a mean ${\text{R}}^{2}$ of 0.998 for CS. TSQP displayed an RMSE of 1.567 MPa (SD = 0.187 MPa) and an average ${\text{R}}^{2}$ of 0.994 (SD = 0.003). DGT obtained an RMSE of 1.354 MPa (SD = 0.165 MPa) and an average ${\text{R}}^{2}$ of 0.996 (SD = 0.002). The mean ${\text{R}}^{2}$ and RMSE for slump flow were 0.984 (SD = 0.004) and 3.233 mm (SD = 0.321 mm) for GBQP, 0.984 (SD = 0.003) and 3.233 mm (SD = 0.298 mm) for TSQP, and 0.989 (SD = 0.003) and 2.987 mm (SD = 0.276 mm) for DGT. High stability throughout the gaps is indicated by these low SD values, which are compiled in Table 10.

**Table 10:** Stability of model performance across tenfold cross-validation.

Model	CS R^2^ mean (SD)	CS RMSE mean (SD, MPa)	Slump R^2^ mean (SD)	Slump RMSE mean (SD, mm)
GBQP	0.998 (0.002)	1.226 (0.154)	0.984 (0.004)	3.233 (0.321)
TSQP	0.994 (0.003)	1.567 (0.187)	0.984 (0.003)	3.233 (0.298)
DGT	0.996 (0.002)	1.354 (0.165)	0.989 (0.003)	2.987 (0.276)

### Feature importance and engineering using SHAP

In order to make clear how input qualities were prioritized in the GBQP, TSQP, and DGT models, the significance of each property was assessed using SHAP (SHApley Additive Description). SHAP uses a dataset of 191 HPC mixtures and rates each feature according to how it affects the predictions of slump flow and CS. Due to their high link with cement hydration and strength development, the research revealed that the most relevant parameters were the water-to-binder ratio (W/B) and silica fume (SF), with mean SHAP values of 0.42 and 0.35 for CS, respectively. With values of 0.18 and 0.15, respectively, superplasticizer (SP) and fly ash (FA) came next, demonstrating their contribution to performance.

Low-impact features (such as coarse aggregate size and SHAP < 0.05) were eliminated during feature selection in order to simplify the model and increase RMSE for GBQP by 5%. Using feature engineering, a composite feature (W/B × SF) was created to capture interaction effects that resulted in a 3% increase in the TSQP drop flow, R^2^. The top characteristics and their SHAP values are compiled in Table 11.

**Table 11:** Feature importance based on SHAP analysis.

Feature	Mean SHAP value CS	Mean SHAP value (Slump)
Water-to-Binder (W/B)	0.42	0.38
Silica Fume (SF)	0.35	0.30
Superplasticizer (SP)	0.18	0.22
Fly Ash (FA)	0.15	0.14

### Limitations of proposed models

Although they have limits, the GBQP, TSQP, and DGT models are excellent at predicting HPC features. First, they might perform poorly when extrapolating outside of the range of the training dataset (for example, silica fume: 0–27 percent, water-to-binder ratio: 19–47%), as might be the case for innovative HPC formulations. Second, since models depend on patterns in the 191-sample dataset, using unusual mix designs with uncommon ingredients (such nanomaterials) may result in decreased accuracy. Third, because GWO and QPSO are sensitive to data quality, performance can be negatively impacted by noisy or missing input data. Lastly, real-time use on devices with limited resources is limited by the computational effort of training (12–17 min per fold, Section “Computational complexity analysis”). To improve reliability, future research should enlarge the dataset, apply thorough preprocessing, and investigate lightweight optimization.

### Practical deployment and data quality considerations

In order to use the suggested models (GBQP, TSQP, and DGT) in actual building situations, practical issues such as data quality control, hardware requirements, and integration with current mix design procedures must be taken into account. By incorporating these models into concrete mix design tools, engineers may enter mix parameters (such as the ratio of water to binder and the dosage of superplasticizer) and get real-time forecasts of slump flow and CS. The inference times (0.8 to 1.5 s per prediction), as indicated in Table 7, are sufficient for on-site decision making with common hardware (e.g., Intel Core i7 with 16 GB of RAM). Cloud-based platforms with GPU capabilities, such AWS EC2, can handle bigger datasets or numerous forecasts at once for scalability, making them suitable for huge projects.

For accurate predictions, data quality is essential. The 191 HPC mixed dataset^38^ was preprocessed by standardizing input ranges, averaging for small gaps, and removing outliers and missing values. Nevertheless, noise in real-world data from building sites, such as inaccurate water content measurements or missing inputs, could impair model performance. Robust preprocessing processes that incorporate feature extraction and outlier identification should be put in place to solve this problem. Important inputs like silica fume and the water-to-binder ratio have high SHAP values, according to the sensitivity analysis in Section “Feature importance and engineering using SHAP”, Table 11, suggesting that precise measurement of these parameters is essential. As mentioned in Section “Impact of real-world environmental conditions”, the models’ capacity to take environmental changes (such as temperature and humidity) into account is limited by their reliance on controlled laboratory data. To improve resilience to actual data quality issues, field data should be taken into account in future research.

By entering mix parameters like water-to-binder ratio and silica fume via an intuitive interface, engineers may utilize the GBQP and TSQP models in their daily concrete mix design to estimate CS and slump flow instantaneously. These models enable quick decisions to be made on the spot with inference times of 0.8 to 1.2 s. GitHub offers a Python-based application that integrates with mix design software and implements these models. The program manages real-time forecasts and needs conventional hardware, including an Intel Core i7 and 16 GB of RAM.

## Conclusion

In order to forecast the CS and slump flow of HPC, this study integrated T-SFIS, GBM, and DT with GWO and QPSO to create the combined models GBQP, TSQP, and DGT. These models outperformed baselines like XGBoost and CatBoost, with GBQP reaching R^2^ = 0.998 and RMSE = 1.226 MPa for CS and TSQP achieving R^2^ = 0.984 and RMSE = 3.233 mm for slump flow. The generalizability was validated by external evaluation on 50 HPC mixes. Despite the short dataset, the application of metaheuristics improved accuracy and successfully caught nonlinear interactions. But there are still restrictions. The risk of overfitting is increased by the short size of the dataset; cross-validation reduces this risk by a factor of 10, but it is not completely removed. The application to real-world situations, where environmental factors like humidity increase variability, is limited by the dependence on controlled laboratory data. Deployment on low-power devices may be hindered by the mixing models’ computational complexity. To increase robustness and empirically test the models, future research should expand the dataset with field-collected HPC mixes. GA and PSO are two more metaheuristics that could be integrated to further optimize performance. The creation of a web-based application that builds upon the Python prototype would boost workflows for mixture design in practice.

## Data Availability

The datasets used and/or analyzed during the current study available from the corresponding author on reasonable request.
